# The reconstruction and biochemical characterization of ancestral genes furnish insights into the evolution of terpene synthase function in the Poaceae

**DOI:** 10.1007/s11103-020-01037-4

**Published:** 2020-07-18

**Authors:** Katrin Luck, Xinlu Chen, Ayla M. Norris, Feng Chen, Jonathan Gershenzon, Tobias G. Köllner

**Affiliations:** 1 Department of Biochemistry, Max Planck Institute for Chemical Ecology, Hans-Knöll Straße 8, 07745 Jena, Germany; 2 Department of Plant Sciences, University of Tennessee, Knoxville, TN 37996 USA; 3 Graduate School of Genome Science and Technology, University of Tennessee, Knoxville, TN 37996 USA

**Keywords:** Terpene synthase, Evolution, Maize, Grasses, Ancestral sequence reconstruction

## Abstract

**Key Message:**

Distinct catalytic features of the Poaceae TPS-a subfamily arose early in grass evolution and the reactions catalyzed have become more complex with time.

**Abstract:**

The structural diversity of terpenes found in nature is mainly determined by terpene synthases (TPS). TPS enzymes accept ubiquitous prenyl diphosphates as substrates and convert them into the various terpene skeletons by catalyzing a carbocation-driven reaction. Based on their sequence similarity, terpene synthases from land plants can be divided into different subfamilies, TPS-a to TPS-h. In this study, we aimed to understand the evolution and functional diversification of the TPS-a subfamily in the Poaceae (the grass family), a plant family that contains important crops such as maize, wheat, rice, and sorghum. Sequence comparisons showed that aside from one clade shared with other monocot plants, the Poaceae TPS-a subfamily consists of five well-defined clades I–V, the common ancestor of which probably originated very early in the evolution of the grasses. A survey of the TPS literature and the characterization of representative TPS enzymes from clades I–III revealed clade-specific substrate and product specificities. The enzymes in both clade I and II function as sesquiterpene synthases with clade I enzymes catalyzing initial C10-C1 or C11-C1 ring closures and clade II enzymes catalyzing C6-C1 closures. The enzymes of clade III mainly act as monoterpene synthases, forming cyclic and acyclic monoterpenes. The reconstruction and characterization of clade ancestors demonstrated that the differences among clades I–III were already present in their ancestors. However, the ancestors generally catalyzed simpler reactions with less double-bond isomerization and fewer cyclization steps. Overall, our data indicate an early origin of key enzymatic features of TPS-a enzymes in the Poaceae, and the development of more complex reactions over the course of evolution.

**Electronic supplementary material:**

The online version of this article (10.1007/s11103-020-01037-4) contains supplementary material, which is available to authorized users.

## Introduction

Terpenes are a structurally diverse group of natural products that are ubiquitous in plants and many other organisms. They are involved in basic physiological processes, but also play important roles in plant–plant and plant–insect interactions. Many plants, for example, produce and release volatile terpenes in response to insect herbivory to attract natural enemies of their herbivores (Unsicker et al. 2009). Infection with plant pathogens can also induce the formation of terpenes, which in turn act as phytoalexins (Block et al. 2019). Until now, more than 40,000 terpenes have been described (Tholl 2015). This structural diversity can mainly be attributed to terpene synthases (TPS), a class of enzymes that catalyze the conversion of prenyl diphosphates into the different terpene skeletons (reviewed in Degenhardt et al. 2009; Karunanithi and Zerbe 2019). Most monoterpene synthases accept the precursor geranyl diphosphate (GPP) as substrate and produce monoterpenes (C_10_), while most sesquiterpene synthases convert (*E*,*E*)-farnesyl diphosphate (FPP) into sesquiterpenes (C_15_), and diterpene synthases catalyze the conversion of geranylgeranyl diphosphate (GGPP) into the different diterpenes (C_20_). Interestingly, a number of terpene synthases have been described to act also on *cis*-prenyl diphosphates such as neryl diphosphate, (*Z*,*Z*)-FPP, and nerylneryl diphosphate (Schilmiller et al. 2009; Sallaud et al. 2009; Zi et al. 2014). The terpenes formed may exert a variety of biological functions (Unsicker et al. 2009; Block et al. 2019). They may also act as substrates for modifying enzymes such as cytochrome P450 monooxygenases, *O*-methyltransferases, and acyltransferases (Dudareva et al. 2004; Degenhardt et al. 2009; Bathe and Tissier 2019).

Although terpene synthases often have broad substrate specificity and accept GPP, FPP, and GGPP in vitro, their *in planta* function may be narrower due to their subcellular localization (Pazouki and Niinemetz 2016). Monoterpene synthases and diterpene synthases typically contain N-terminal signal peptides and are transported into plastids, the site for GPP and GGPP, but not FPP production. Sesquiterpene synthases, however, are usually found in the cytosol, a site of FPP, but not GPP nor GGPP production (Degenhardt et al. 2009). There is increasing evidence for an exchange of prenyl diphosphates between the different compartments of a plant cell, especially under stress conditions (e.g. Gutensohn et al. 2013; Dong et al. 2016). Thus, the subcellular localization of TPS enzymes as well as the exchange of TPS substrates between compartments can determine the *in planta* function of terpene synthases with broad substrate specificity (Pazouki and Niinemetz 2016).

Based on sequence similarity and phylogenetic relationships, terpene synthases can be divided into eight subfamilies, TPS-a to TPS-h (Bohlmann et al. 1998; Chen et al. 2011). The subfamilies TPS-a, TPS-b, and TPS-g comprise predominantly monoterpene synthases and sesquiterpene synthases of angiosperms, while the gymnosperm monoterpene and sesquiterpene synthases belong to the TPS-d subfamily. The subfamilies TPS-c and TPS-e/f mainly comprise diterpene synthases from angiosperms and gymnosperms, and the subfamily TPS-h contains diterpene synthases specific for the Lycopodiophyta (clubmosses) (Bohlmann et al. 1998; Chen et al. 2011; Karunanithi and Zerbe 2019). Angiosperm plants typically possess 20–60 *TPS* genes, most of which belong to the *TPS-a* subfamily (Chen et al. 2011).

The reaction mechanism catalyzed by terpene synthases starts with the formation of a carbocation intermediate that is either derived by a metal ion-dependent ionization of the prenyl diphosphate substrate (class I TPS) or by protonation of a double bond in the prenyl diphosphate tail (class II TPS) (Degenhardt et al. 2009). While most if not all monoterpene synthases and sesquiterpene synthases belong to class I TPS, diterpene synthases can be found in both classes. After its formation, the carbocation intermediate can undergo a complex cascade of different cyclizations, hydride shifts, and skeleton rearrangements until the reaction is terminated by a deprotonation or addition of a nucleophile such as water (Degenhardt et al. 2009). Many terpene synthases are multiproduct enzymes that produce complex mixtures of compounds (Steele et al. 1998; Garms et al. 2010; Irmisch et al. 2014). However, the individual components of the mixture often share the same terpene skeleton or at least belong to structurally related skeleton types, the formation of which is mainly determined by the first cyclization of the reaction cascade (e.g. Köllner et al. 2004; Garms et al. 2010; Irmisch et al. 2012). The maize sesquiterpene synthase TPS4, for example, produces a mixture of more than 22 compounds that are almost all derived by an initial C6-C1 ring closure (numbering as for FPP) of the (*Z*,*E*)-farnesyl carbocation (Köllner et al. 2004). In contrast, all products formed by MrTPS2, a terpene synthase found in *Matricaria recutita*, are derived by an initial C11-C1 closure of the (*E*,*E*)-farnesyl carbocation (Irmisch et al. 2012; Hong et al. 2013). Although the basic phylogeny of the TPS family, including division into subfamilies, is well accepted, evolutionary relationships among many of the reaction types are still not well understood.

As part of our ongoing effort to investigate the formation of terpenes in the grass family (Poaceae), we aimed to understand the evolution of catalytic function in the *TPS-a* enzymes of this plant family. In the Poaceae, the *TPS-a* subfamily dominates the *TPS* gene family in terms of absolute numbers of genes (Chen et al. 2011) and includes a large number of previously characterized TPS enzymes. Ancestral sequences of the Poaceae *TPS-a* subfamily were reconstructed, synthesized, and heterologously expressed in *Escherichia coli*. Recombinant enzymes were incubated with the potential substrates GPP and (*E*,*E*)-FPP, and reaction products were analyzed using gas chromatography-mass spectrometry (GC–MS). Our data showed that many of the distinct features of specific clades of the Poaceae *TPS-a* subfamily arose early in grass evolution, but the reactions catalyzed have become more complex with time.

## Results

### Most of the *TPS-a* genes in the Poaceae belong to five well-defined clades

**Fig. 1:**
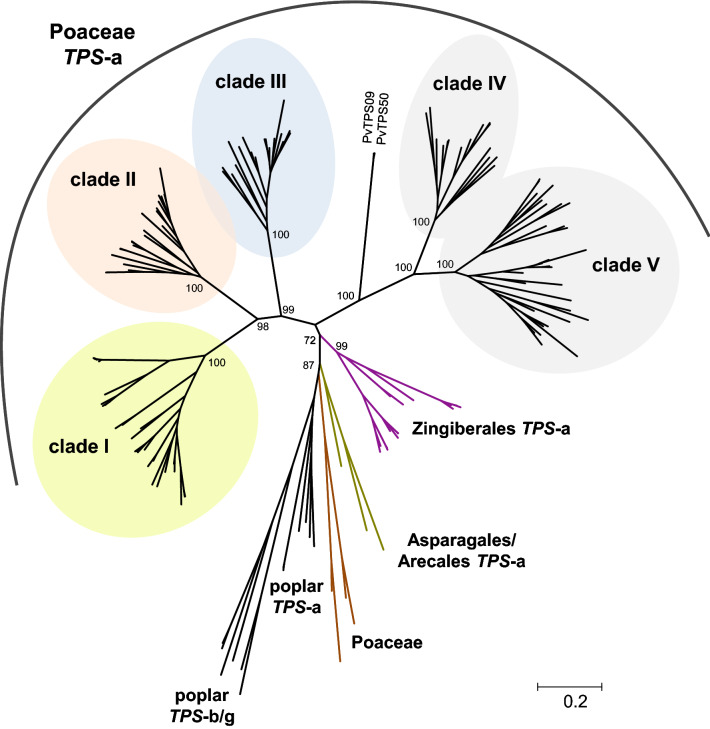
Dendrogram analysis of terpene synthase genes from six Poaceae species (*Zea mays*, *Sorghum bicolor*, *Setaria italica*, *Panicum virgatum*, *Oryza sativa*, *Brachypodium distachyon*) and from diverse species of Arecales, Asparagales, and Zingiberales. The tree was inferred by using the Maximum Likelihood method based on the General Time Reversible model (Rates among sites, G + I). Bootstrap values (n = 1000 replicates) are shown next to each node. The tree is drawn to scale, with branch lengths measured in the number of substitutions per site. Terpene synthases from *Populus trichocarpa* were included as an outgroup

In order to identify *TPS-a* genes from the Poaceae, we constructed a phylogenetic tree of terpene synthase genes that were extracted using BLASTP analysis from six grass species, including *Zea mays*, *Sorghum bicolor*, *Setaria italica*, *Panicum virgatum*, *Oryza sativa*, and *Brachypodium distachyon*, and a number of other monocotyledonous and dicotyledonous species available in the NCBI database and the Phytozome 9.1 database (Supplemental Figure S1). In accordance with the literature (Chen et al. 2011), all obtained *TPS* genes clustered into one of the established TPS subfamilies *TPS-a*, *TPS-b*, *TPS-c*, *TPS-e*, *TPS-f*, and *TPS-g*. The *TPS-a* genes formed two groups, one containing genes from dicotyledonous species and the other containing genes of monocotyledonous species (Supplemental Figure S1, Chen et al. 2011). A deeper dendrogram analysis of the identified monocotyledonous *TPS-a* genes revealed five well-defined monophyletic clades, I–V, each of which possessed exclusively *TPS-a* genes from the Poaceae (Fig. 1). Two genes from *P*.* virgatum* were similar to clade IV and clade V sequences, but clustered separately. Four other Poaceae *TPS-a* genes, from rice, sorghum, and bamboo, were found to form a very basal clade among the monocotyledonous *TPS-a* subfamily, while *TPS* genes from families outside the Poaceae clustered separately from the five Poaceae clades (Fig. 1).

### The Poaceae *TPS-a* genes in clades I–V encode enzymes with distinct catalytic features

**Fig. 2:**
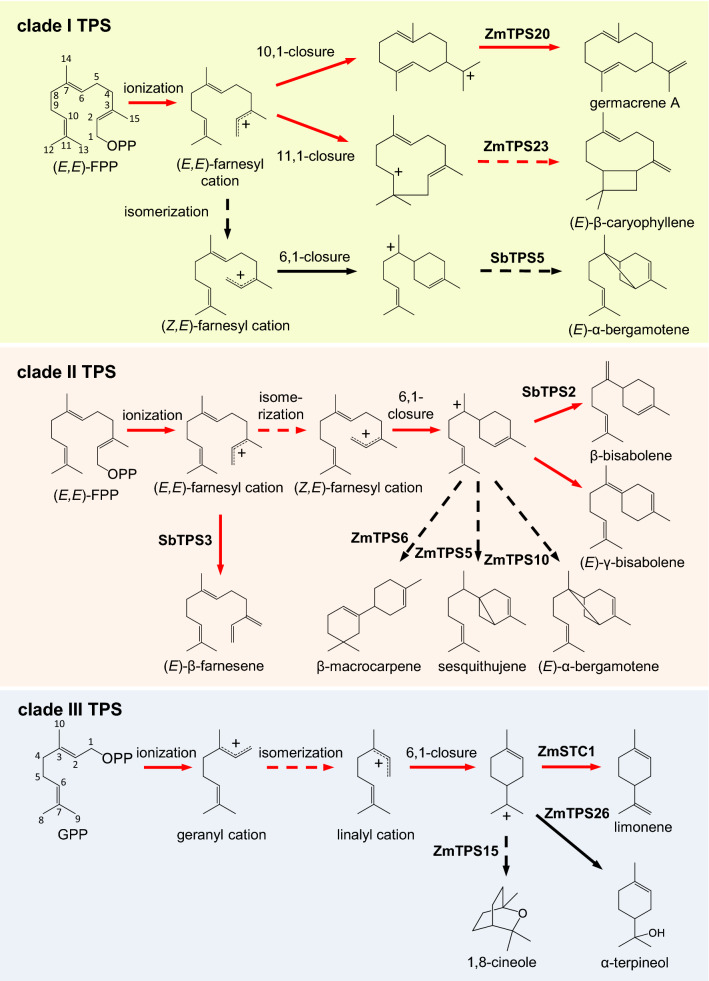
Reactions catalyzed by representative TPS enzymes from clades I–III and their respective ancestors including major carbocationic intermediates. Reactions catalyzed by the putative ancestors are marked with red arrows. Dashed arrows indicate multiple reaction steps

A literature survey of the properties of the Poaceae *TPS-a* encoded enzymes characterized to date revealed that clades I–V have distinctive catalytic features (Table 1). All previously characterized clade I enzymes are sesquiterpene synthases that accept (*E*,*E*)-FPP as substrate and, with two exceptions, catalyze an initial C10-C1 or C11-C1 closure (Fig. 2). All characterized clade II enzymes are also sesquiterpene synthases, but produce either acyclic terpenes or cyclic terpenes derived from an initial C6-C1 closure. In contrast to the sesquiterpene synthases of clades I and II, characterized clade III enzymes mainly accept GPP as substrate and produce monoterpenes derived from an initial C6-C1 closure (Fig. 2). Moreover, most of the clade III sequences are predicted to contain a transit peptide for plastid localization, indicating that these enzymes likely function as monoterpene synthases in planta. The terpene synthases in clades IV and V, however, are diverse in terms of substrate specificity and initial cyclization reaction. The characterized enzymes in these clades form monoterpenes or sesquiterpenes, and catalyze initial C6-C1, C10-C1, or C11-C1 closures (Table 1).

**Table 1:** Characterized TPS-a enzymes in the Poaceae

Clade	TPS Name	Species	Grass lineage	Main product(s)	Initial cyclization	References
I	Bradi3g14710	*Brachypodium distachyon*	BEP	(*E*)-β-Caryophyllene	11–1	This study
I	ZS	*Chrysopogon zizanoides*	PACMAD	(+)-Zizaene	6–1	Hartwig et al. (2015)
I	ObTPS1	*Oryza barthii*	BEP	(*E*)-β-Caryophyllene	11–1	Chen et al. (2014)
I	OgTPS1	*Oryza glaberrima*	BEP	(*E*)-β-Caryophyllene/Germacrene A	11–1/10–1	Chen et al. (2014)
I	OgluTPS1	*Oryza glumaepatula*	BEP	(*E*)-β-Caryophyllene	11–1	Chen et al. (2014)
I	OnTPS1	*Oryza nivara*	BEP	(*E*)-β-Caryophyllene	11–1	Chen et al. (2014)
I	OoTPS1	*Oryza officinalis*	BEP	(*E*)-β-Caryophyllene/Germacrene A	11–1/10–1	Chen et al. (2014)
I	OrTPS1	*Oryza rufipogon*	BEP	Germacrene A/Germacrene D	10–1	Chen et al. (2014)
I	OsTPS1	*Oryza sativa*	BEP	(*E*)-β-Caryophyllene/Germacrene A	11–1	Yuan et al. (2008)
I	PvTPS1	*Panicum virgatum*	PACMAD	Cycloisosativene	10–1	Muchlinski et al. (2019)
I	PvTPS11	*Panicum virgatum*	PACMAD	(*E*)-β-Caryophyllene	11–1	Muchlinski et al. (2019)
I	PvTPS14	*Panicum virgatum*	PACMAD	(*E*)-β-Caryophyllene	11–1	Muchlinski et al. (2019)
I	PvTPS19	*Panicum virgatum*	PACMAD	(*E*)-β-Caryophyllene	11–1	Muchlinski et al. (2019)
I	SbTPS4	*Sorghum bicolor*	PACMAD	(*E*)-β-Caryophyllene	11–1	Zhuang et al. (2012)
I	SbTPS5	*Sorghum bicolor*	PACMAD	(*Z*)-γ-Bisabolene/(*E*)-α-Bergamotene	6–1	Zhuang et al. (2012)
I	ZmTPS23-Del	*Zea mays*	PACMAD	(*E*)-β-Caryophyllene	11–1	Köllner et al. (2008a)
I	ZmTPS20-Del	*Zea mays*	PACMAD	Germacrene A	10–1	This study
I	ZmTPS22-Del	*Zea mays*	PACMAD	β-Copaene	10–1	This study
II	Bradi3g15956	*Brachypodium distachyon*	BEP	β-Macrocarpene/γ-Macrocarpene	6–1	This study
II	Oba080	*Oryza barthii*	BEP	(*E*)-β-Farnesene/Zingiberene	no/6–1	Chen et al. (2020)
II	Og100	*Oryza glaberrima*	BEP	Zingiberene	6–1	Chen et al. (2020)
II	On080	*Oryza nivara*	BEP	(*E*)-β-Farnesene	no	Chen et al. (2020)
II	Or100	*Oryza rufipogon*	BEP	β-Bisabolene/ β-Sesquiphellandrene	6–1	Chen et al. (2020)
II	Os100	*Oryza sativa*	BEP	β-Bisabolene/ β-Sesquiphellandrene	6–1	Yuan et al. (2008)
II	Os120	*Oryza sativa*	BEP	β-Bisabolene	6–1	Chen et al. (2020)
II	PvTPS16	*Panicum virgatum*	PACMAD	(*E*)-β-Farnesene	no	Muchlinski et al. (2019)
II	PvTPS17	*Panicum virgatum*	PACMAD	β-Bisabolene	6–1	Muchlinski et al. (2019)
II	PvTPS20	*Panicum virgatum*	PACMAD	β-Bisabolene	6–1	Muchlinski et al. (2019)
II	PvTPS69	*Panicum virgatum*	PACMAD	(*E*)-β-Farnesene	no	Muchlinski et al. (2019)
II	PvTPS94	*Panicum virgatum*	PACMAD	α-Santalene	6–1	Muchlinski et al. (2019)
II	PvTPS109	*Panicum virgatum*	PACMAD	(*E*)-β-Farnesene	no	Muchlinski et al. (2019)
II	SbTPS1	*Sorghum bicolor*	PACMAD	Zingiberene	6–1	Zhuang et al. (2012)
II	SbTPS2	*Sorghum bicolor*	PACMAD	β-Sesquiphellandrene	6–1	Zhuang et al. (2012)
II	SbTPS3	*Sorghum bicolor*	PACMAD	(*E*)-β-Farnesene/(*E*)-α-Bergamotene	no/6–1	Zhuang et al. (2012)
II	ZmTPS4-B73	*Zea mays*	PACMAD	7-*epi*-Sesquithujene/β-Bisabolene	6–1	Köllner et al. (2004)
II	ZmTPS5-Del1	*Zea mays*	PACMAD	Sesquithujene/β-Bisabolene	6–1	Köllner et al. (2004)
II	ZmTPS6-B73	*Zea mays*	PACMAD	β-Macrocarpene	6–1	Köllner et al. (2008b)
II	ZmTPS11-B73	*Zea mays*	PACMAD	β-Macrocarpene	6–1	Köllner et al. (2008b)
II	ZmTPS10-B73	*Zea mays*	PACMAD	(*E*)-β-Farnesene/(*E*)-α-Bergamotene	no/6–1	Schnee et al. (2006)
II	ZmTPS10-dip	*Zea diploperennis*	PACMAD	(*E*)-β-Farnesene/(*E*)-α-Bergamotene	no/6–1	Köllner et al. (2009)
II	ZmTPS10-hue	*Zea huehuetenangensis*	PACMAD	(*E*)-β-Farnesene/(*E*)-α-Bergamotene	no/6–1	Köllner et al. (2009)
II	ZmTPS10-mex	*Zea mexicana*	PACMAD	(*E*)-β-Farnesene/(*E*)-α-Bergamotene	no/6–1	Köllner et al. (2009)
II	ZmTPS10-per	*Zea perennis*	PACMAD	(*E*)-β-Farnesene/(*E*)-α-Bergamotene	no/6–1	Köllner et al. (2009)
III	PvTPS03	*Panicum virgatum*	PACMAD	(*E*)-γ-Bisabolene	6–1	Muchlinski et al. (2019)
III	PvTPS04	*Panicum virgatum*	PACMAD	α-Terpinolene	6–1^MT^	Muchlinski et al. (2019)
III	PvTPS08	*Panicum virgatum*	PACMAD	1,8-Cineole	6–1^MT^	Muchlinski et al. (2019)
III	PvTPS36	*Panicum virgatum*	PACMAD	Limonene	6–1^MT^	Muchlinski et al. (2019)
III	PvTPS83	*Panicum virgatum*	PACMAD	(*E*)-γ-Bisabolene	6–1	Muchlinski et al. (2019)
III	ZmTPS15-B73	*Zea mays*	PACMAD	1,8-Cineole	6–1^MT^	This study
III	ZmTPS19-B73	*Zea mays*	PACMAD	α-Terpineol/γ-Terpinene	6–1^MT^	Lin et al. (2008)
III	ZmTPS26-B73	*Zea mays*	PACMAD	α-Terpineol/γ-Terpinene	6–1^MT^	Lin et al. (2008)
IV	PvTPS05	*Panicum virgatum*	PACMAD	α-Selinene	10–1	Muchlinski et al. (2019)
IV	PvTPS56	*Panicum virgatum*	PACMAD	α-Terpineol	6–1^MT^	Muchlinski et al. (2019)
IV	ZmEDS	*Zea mays*	PACMAD	Eudesmane-2,11-diol	10–1	Liang et al. (2018)
IV	ZmTPS21	*Zea mays*	PACMAD	β-Selinene	10–1	Ding et al. (2017)
V	OsTPS3	*Oryza sativa*	BEP	(*E*,*E*)-Farnesol	no	Cheng et al. (2007)
V	OsTPS19	*Oryza sativa*	BEP	Limonene	6–1^MT^	Chen et al. (2018)
V	OsTPS20	*Oryza sativa*	BEP	Limonene	6–1^MT^	Chen et al. (2018)
V	PvTPS06	*Panicum virgatum*	PACMAD	δ-Cadinene	10–1	Muchlinski et al. (2019)
V	PvTPS53	*Panicum virgatum*	PACMAD	Geraniol	no^MT^	Muchlinski et al. (2019)
V	PvTPS54	*Panicum virgatum*	PACMAD	Not active	–	Muchlinski et al. (2019)
V	PvTPS55	*Panicum virgatum*	PACMAD	Germacrene D	10–1	Muchlinski et al. (2019)
V	PvTPS101	*Panicum virgatum*	PACMAD	Geraniol	no^MT^	Muchlinski et al. (2019)
V	TaPS	*Triticum aestivum*	PACMAD	β-Patchoulene	11–1	Pu et al. (2019)
V	ZmTPS7	*Zea mays*	PACMAD	τ-Cadinol	10–1	Ren et al. (2016)
V	ZmTPS8	*Zea mays*	PACMAD	Germacrene D	10–1	Fontana et al. (2011)

*MT* monoterpene synthase activity

### Characterization of TPS-a enzymes from maize and *Brachypodium distachyon*

**Fig. 3:**
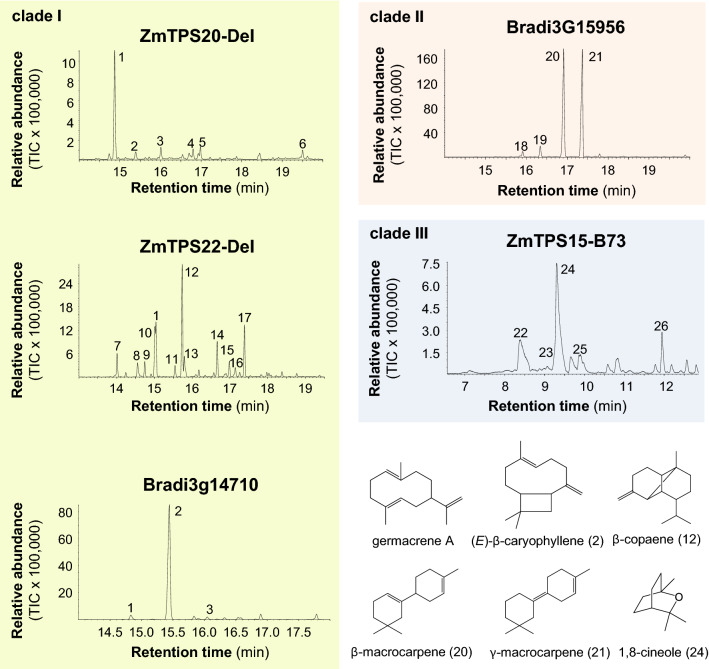
Characterization of selected terpene synthases from the Poaceae TPS-a clades I–III. Genes were heterologously expressed in *Escherichia coli* and partially purified proteins were incubated with (*E*,*E*)-FPP (TPS20-Del, TPS22-Del, Bradi3g14710, Bradi3g15956) or GPP (TPS15-B73). TPS reaction products were collected from the headspace of the enzyme assays using a solid phase microextraction (SPME) fiber and analyzed by gas chromatography-mass spectrometry. Total ion current chromatograms are shown. 1, β-elemene; 2, (*E*)-β-caryophyllene; 3, α-humulene; 4, unidentified sesquiterpene hydrocarbon1; 5, unidentified sesquiterpene hydrocarbon2; 6, unidentified sesquiterpene alcohol1; 7, δ-elemene; 8, unidentified sesquiterpene hydrocarbon3; 9, α-copaene; 10, β-cubebene; 11, β-ylangene; 12, β-copaene; 13, γ-elemene; 14, germacrene D; 15, α-muurolene; 16, cubebol; 17, δ-cadinene; 18, (*E*)-β-farnesene; 19, unidentified sesquiterpene hydrocarbon4; 20, β-macrocarpene; 21, γ-macrocarpene; 22, myrcene; 23, limonene; 24, 1,8-cineole; 25, (*E*)-β-ocimene; 26, linalool. Structures of major TPS products are shown

To provide more information about the differences among these Poaceae *TPS-a* clades, we characterized three previously unstudied *TPS-a* genes from maize and two *TPS-a* genes from *B*.* distachyon*, a species from which no TPS has been characterized so far. The selected terpene synthases belong to clade I (maize ZmTPS20-Del and ZmTPS22-Del, *B*.* distachyon* Bradi3g14710), clade II (*B*.* distachyon* Bradi3g15956), and clade III (maize ZmTPS15-B73). Genes were cloned and heterologously expressed in *E*.* coli*, and partially purified proteins were incubated with the potential substrates GPP and (*E*,*E*)-FPP in the presence of 10 mM Mg^2+^ as cofactor. Enzyme products were analyzed using GC–MS. The three clade I enzymes ZmTPS20-Del, ZmTPS22-Del, and Bradi3g14710 showed exclusively sesquiterpene synthase activity and produced sesquiterpenes derived by either an initial C10-C1 closure or an initial C11-C1 closure, corresponding to the properties of clade I enzymes in the literature. ZmTPS20-Del produced mainly germacrene A (C10-C1 closure), which appeared as the thermal rearrangement product β-elemene in the traces of the GC chromatograms (Fig. 3; Supplemental Figure S2). ZmTPS22-Del produced a complex mixture of sesquiterpenes with the bicyclic β-copaene (C10-C1 closure) as the major component (Fig. 3). In contrast to ZmTPS20-Del and ZmTPS22-Del, Bradi3g14710 had higher product specificity and formed (*E*)-β-caryophyllene (C11-C1 closure) with trace amounts of α-humulene and germacrene A (Fig. 3). Monoterpene synthase activity with GPP was not observed for any of the three enzymes. The clade II enzyme Bradi3g15956 converted (*E*,*E*)-FPP into β-macrocarpene and γ-macrocarpene, two bicyclic sesquiterpenes both derived by an initial C6-C1-closure (Fig. 3). GPP, however, was not accepted by Bradi3g15956 as substrate. Maize ZmTPS15-B73 showed no activity when provided with (*E*,*E*)-FPP. However, it converted GPP into a mixture of monoterpenes dominated by the bicyclic alcohol 1,8-cineole (C6-C1 closure) (Fig. 3). Subcellular localization prediction using three different algorithms revealed the presence of an N-terminal transit peptide for plastid localization (Supplemental Figure S3), indicating that ZmTPS15-B73 likely acts as a monoterpene synthase in maize plastids.

### The reconstruction and characterization of TPS-a ancestor enzymes

**Fig. 4:**
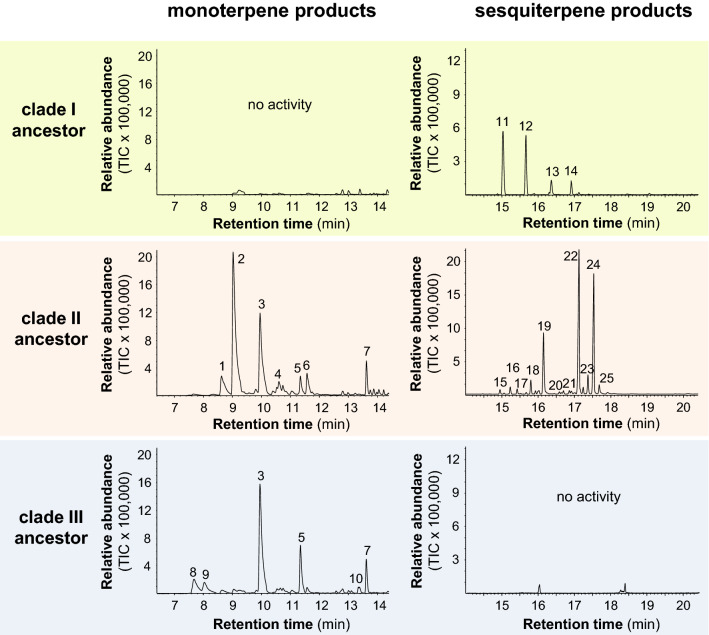
Proteins encoded by putative ancestors to Poaceae *TPS-a* clades have terpene synthase activity in vitro. Reconstructed *TPS* ancestor sequences of clades I, II, and III of the Poaceae *TPS*-*a* genes were synthesized, cloned, and heterologously expressed in *Escherichia coli*. Partially purified proteins were incubated with the potential substrates GPP and (*E*,*E*)-FPP. TPS reaction products were collected from the headspace of the enzyme assays using a solid phase microextraction (SPME) fiber and analyzed by gas chromatography-mass spectrometry. Total ion current chromatograms are shown. 1, β-phellandrene; 2, myrcene; 3, limonene; 4, (*E*)-β-ocimene; 5, terpinolene; 6, linalool; 7, α-terpineol; 8, α-pinene; 9, camphene; 10, terpinen-4-ol; 11, β-elemene; 12; (*E*)-β-caryophyllene; 13, α-humulene; 14, germacrene D; 15, 7-*epi*-sesquithujene; 16, sesquithujene; 17, (*Z*)-α-bergamotene; 18, (*E*)-α-bergamotene; 19, (*E*)-β-farnesene; 20, sesquisabinene; 21, zingiberene; 22, β-bisabolene; 23, β-sesquiphellandrene; 24, (*E*)-γ-bisabolene; 25, (*Z*)-α-bisabolene

Most of the enzymes in clades I–III share clade-specific catalytic features, catalyzing either a common initial cyclization or having the same substrate specificity (Table 1; Fig. 2). To test whether these features were already pronounced in the ancestors of the different clades, we reconstructed the respective ancestor sequences, expressed the synthesized genes in *E*.* coli*, and tested the recombinant enzymes obtained with GPP and (*E*,*E*)-FPP. The putative clade I ancestor showed only sesquiterpene synthase activity and produced mainly germacrene A (C10-C1) and (*E*)-β-caryophyllene (C11-C1) (Figs. 2 and 4, Supplemental Figure S2). The putative clade II ancestor accepted both GPP and (*E*,*E*)-FPP as substrate and produced mixtures of monoterpenes and sesquiterpenes. While the monoterpene blend was dominated by the acyclic myrcene, the major sesquiterpenes were identified as β-bisabolene and (*E*)-γ-bisabolene, which are both formed via an initial C6-C1 closure (Figs. 2 and 4). In contrast to the putative ancestors of clade I and clade II, the clade III ancestor, which was expressed without the N-terminal signal peptide, possessed exclusively monoterpene synthase activity and produced the cyclic limonene (C6-C1) as its major product (Figs. 2 and 4).

## Discussion

Many important crops such as maize, wheat, rice, and sorghum belong to the Poaceae, a plant family known to be rich in terpenes. Terpenes play important roles in plant defense and their biosynthesis has been intensively investigated during the last three decades (Degenhardt et al. 2009). In this study, we aimed to understand the functional evolution of the TPS-a subfamily in the grasses by reconstructing and characterizing ancestral TPS enzymes and comparing their catalytic activities to those of modern terpene synthases.

**Fig. 5:**
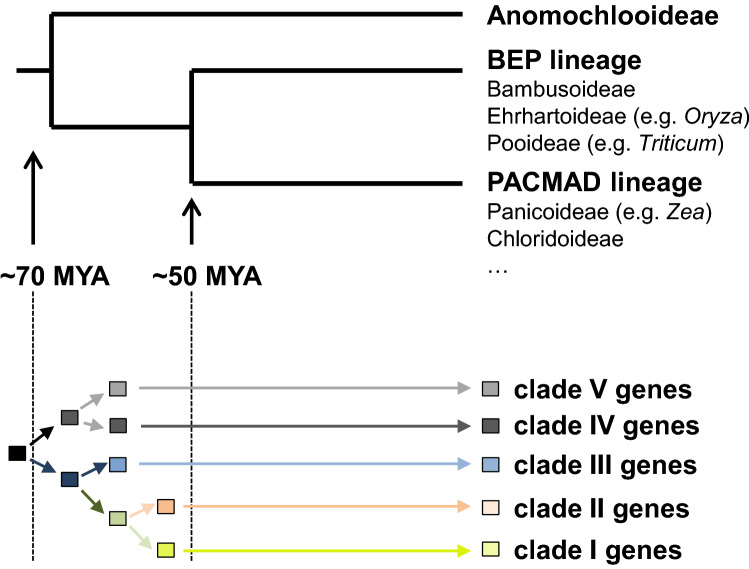
Model for the evolution of the five different *TPS-a* clades in the Poaceae. The phylogenetic relationships of the clades from Fig. 1 are shown together with their alignment to a simplified grass phylogeny. The ancestors of the *TPS-a* clades I–V all evolved through gene duplications before the split of the PACMAD and BEP lineages

The Poaceae family consists of three major lineages, the PACMAD (Panicoideae, Arundinoideae, Chloridoideae, Micrairoideae, Aristidoideae, Danthonioideae) lineage, the BEP (Bambusoideae, Ehrhartoideae, Pooideae) lineage, and the basal Anomochlooideae (Strömberg 2011, Fig. 5). Fossil records indicate that the common ancestor of the grasses existed more than 70 Ma in the Late Cretaceous, and that the PACMAD and BEP lineages evolved between 70 and 50 Ma (Strömberg 2011). A phylogenetic analysis of *TPS-a* genes from different monocotyledonous species revealed five monophyletic clades I–V that exclusively contain genes from the Poaceae (Fig. 1). Since all of the five TPS-a clades contain both PACMAD and BEP genes (Table 1; Supplemental Figure S4), it is likely that the direct ancestors of the five clades evolved by at least four consecutive gene duplication events from a common *TPS-a* progenitor prior to the separation of the two lineages (Fig. 5).

A survey of the TPS literature and characterization of representative TPS in this study revealed that most of the enzymes in clades I, II, IV, and V are sesquiterpene synthases, while clade III possesses predominantly monoterpene synthases (Table 1; Fig. 3). It is thus tempting to speculate that the TPS-a progenitor in the early grasses was a sesquiterpene synthase. In this scenario, the ancestor of clade III had to evolve monoterpene synthase activity, presumably by the accumulation of mutations leading to a decreased active site pocket that no longer allowed binding of the FPP substrate, and gained an N-terminal signal peptide for transport to the plastid, where GPP is produced. Indeed, the reconstruction of this ancestor based on the modern clade III sequences and its functional characterization showed that the enzyme was able to convert GPP into monoterpenes but not (*E*,*E*)-FPP into sesquiterpenes (Fig. 4). Interestingly, a few enzymes in clades IV and V have also been described as monoterpene synthases (Table 1), indicating multiple evolution of this enzymatic function within the TPS-a subfamily. OsTPS19 and OsTPS20 from rice, for example, belong to clade V and were found to be localized in plastids. The manipulation of *OsTPS19* gene expression in transgenic rice clearly confirmed its function as a monoterpene synthase *in planta* (Chen et al. 2018). However, the subcellular localization of other putative monoterpene synthases in clades IV and V is rather unclear. Some of them do not appear to contain signal peptides (Muchlinski et al. 2019) and, in contrast to clade III monoterpene synthases that only accept GPP as substrate (Lin et al. 2008; Muchlinski et al. 2019; Fig. 3), they often display both monoterpene and sesquiterpene synthase activity in vitro. Thus, a reliable prediction of their function in vivo is still not possible.

The Poaceae *Tps-a* clades differ among each other in the type of reactions that they catalyze. Nearly all monoterpene and sesquiterpene synthases are class I TPS enzymes, which catalyze a metal ion-dependent ionization of the substrate by abstracting the pyrophosphate moiety (Degenhardt et al. 2009; Fig. 2). The first reaction intermediate formed from GPP by monoterpene synthases is the geranyl cation. It can be deprotonated or captured by water addition to form acyclic monoterpenes, but due to conformational constraints can only be cyclized after isomerization to the linalyl cation, C1 of which is located near to the C6-C7 double bond (Fig. 2). The ionization of (*E*,*E*)-FPP by sesquiterpene synthases results in the formation of the (*E*,*E*)-farnesyl cation, which can undergo direct C10-C1 or C11-C1 closures to yield 10-membered or 11-membered rings, respectively. Alternatively, the isomerization of the C2–C3 double bond of the (*E*,*E*)-farnesyl cation leads to the tertiary (*Z*,*E*)-farnesyl cation, in direct analogy with the isomerization of GPP to the linalyl cation. The (*Z*,*E*)-farnesyl cation can then undergo cyclization involving either the central or distal double bond forming 1,6-, 1,7-, 1,10-, or 1,11- products (Degenhardt et al. 2009). Although the cyclic mono- and sesquiterpene cations formed in this way can be modified by further ring closures, ring contractions, and other rearrangements, the basic skeleton type is determined by the initial cyclization and isomerization reactions (Degenhardt et al. 2009). Interestingly, the different TPS-a enzymes in clades I, II, and III catalyze distinct and clade-specific initial reaction steps. With one exception, all clade I enzymes characterized to date produce sesquiterpenes derived from an initial C10-C1 or C11-C1 closure that does not require isomerization of the farnesyl cation (Fig. 2; Table 1). In contrast, clade II and clade III enzymes catalyze the isomerization of the farnesyl cation and the geranyl cation, respectively, prior to a C6-C1 ring closure. The reconstructed ancestors of clades I, II, and III were shown to catalyze the same basic initial reactions as the present day members of the clade (Figs. 2 and 4), indicating that the key enzymatic features of the modern TPS-a enzymes evolved early in the evolution of the grasses. However, in contrast to the modern enzymes, the reconstructed TPS ancestors formed simpler structures that are mainly derived by a deprotonation of the first cyclic cation (Fig. 2). Present day enzymes typically catalyze reaction cascades with additional steps, such as the isomerization of carbon–carbon double bond in the initial cation to allow alternate ring closures or additional cyclizations. The structural diversity of terpenes formed in today’s grasses likely evolved later in the grass evolution within single subfamilies, genera, or even species. Successive gene duplications of the respective clade ancestors and the subsequent accumulation of mutations led to the multitude of modern TPS-a enzymes, many of which catalyze more complex reactions than the ancestor. Although it is universally accepted that evolution of natural product biosynthesis has led to the formation of more and more complex structures, this process has rarely been documented at the level of a specific enzyme and plant group.

## Materials and methods

### Plant material

Seeds of the maize (*Zea mays* L.) inbred line B73 were provided by KWS seeds (Einbeck, Germany), and seeds of the maize hybrid variety Delprim were obtained from Delley Samen und Pflanzen (Delley, Switzerland). Plants were grown in commercially available potting soil in a climate-controlled chamber with a 16 h photoperiod, 1 mmol (m^2^)^−1^ s^−1^ of photosynthetically-active radiation, a temperature cycle of 22 ºC/18 ºC (day/night), and 65% relative humidity. Ten day old seedlings were harvested and immediately frozen in liquid nitrogen. Seeds of the *Brachypodium distachyon* inbred diploid accession Bd21.3 were obtained from the National Plant Germplasm System, US Department of Agriculture. Seeds were sowed into pots and placed at 4 °C in the dark for 2 days, and then the pots were transferred into a Percival growth chamber. Plants were grown under 16 h light/8 h darkness at 26 °C. Four-week old plants were harvested and immediately frozen in liquid nitrogen.

### Gene identification, sequence analysis, and dendrogram analysis

Using poplar TPS9 (Irmisch et al. 2014) as a query, we conducted a TBLASTN analysis against 6 Poaceae gene sets (*Zea mays*, *Sorghum bicolor*, *Setaria italica*, *Panicum virgatum*, *Oryza sativa*, *Brachypodium distachyon*) and 27 angiosperm gene sets (*Aquilegia coerulea*, *Arabidopsis lyrata*, *A*.* thaliana*, *Brassica rapa*, *Capsella rubella*, *Carica papaya*, *Citrus clementina*, *Citrus x sinensis*, *Cucumis sativus*, *Eucalyptus grandis*, *Eutrema salsugineum*, *Fragaria vesca*, *Glycine max*, *Gossypium raimondii*, *Linum usitatissimum*, *Malus x domestica*, *Manihot esculenta*, *Medicago truncatula*, *Mimulus guttatus*, *Phaseolus vulgaris*, *Populus trichocarpa*, *Prunus persica*, *Ricinus communis*, S*olanum lycopersicum*, *S*.* tuberosum*, *Theobroma cacao*, *Vitis vinifera*) available in the Phytozome 9.1 database (phytozome.jgi.doe.gov). In addition, the NCBI database (www.ncbi.nlm.nih.gov) was similarly screened for *TPS* genes from monocotyledonous species outside the Poaceae (species of Arecales, Asparagales, Liliales, and Zingiberales). Sequences longer than 1499 bp were considered as full-length genes and subjected to phylogenetic analysis. For the estimation of a phylogenetic tree, we used the MUSCLE (codon) algorithm (gap open, − 2.9; gap extend, 0; hydrophobicity multiplier, 1.2; clustering method, UPGMB) implemented in MEGA6 (Tamura et al. 2011) to compute a nucleotide codon alignment with the 932 obtained full-length *TPS* sequences. Based on the MUSCLE alignment, the tree was reconstructed with MEGA6 using a maximum likelihood algorithm (model/method, General Time Reversible model; substitutions type, nucleotide; rates among sites, gamma distributed with invariant sites (G + I); gamma parameters, 5; gaps/missing data treatment, partial deletion; site coverage cutoff, 50%). Guided by the resulting *TPS* tree, 163 out of the 932 sequences could be identified as monocot-specific *TPS-a2* genes and were subjected to deeper phylogenetic analysis using the same methods and parameters as described above, except with a site coverage cutoff of 80%. A bootstrap resampling analysis with 1000 replicates was performed to evaluate the topology of the tree. FASTA files are available as Supplemental data set 1 (angiosperm TPS) and Supplemental data set 2 (Poaceae TPS).

### Ancestor reconstruction

Codon alignments of characterized *TPS-a* genes from the clades I, II, and III were created using the PRANK algorithm provided by the Guidance web-based interface (https://guidance.tau.ac.il/) (MSA scores are given in Supplemental Table S1; FASTA files are available as Supplemental data sets 3 (clade I), 4 (clade II), and 5 (clade III)). The PRANK alignments were loaded into MEGA6 and model tests were performed to search for the optimal substitution models and parameters. Maximum likelihood (ML) trees (Supplemental Figures S5-S7) were generated with MEGA6 using the models and parameters given in Supplemental Table S1. All sites of the sequences were considered for the generation of the trees. The most likely protein ancestor sequences were extracted from the ML trees using the program ExtAncSeqMEGA (Hall 2011) (for accuracy scores see Supplemental Table S1).

### Signal peptide prediction

Signal peptides were predicted with the programs ChloroP (https://www.cbs.dtu.dk/services/ChloroP/), TargetP (https://www.cbs.dtu.dk/services/TargetP/), and Predotar v1.04 (https://urgi.versailles.inra.fr/predotar/).

### Cloning and gene synthesis

Total RNA was isolated using the RNeasy Plant Mini Kit (Qiagen, Hilden, Germany) according to the manufacturer’s instructions. Single-stranded cDNA was synthesized from total RNA using SuperScript™ III Reverse Transcriptase (Invitrogen, Carlsbad, CA) and oligo(dT) primers. The complete open reading frames (ORF) of *ZmTPS20-Del*, *ZmTPS22-Del*, and *ZmTPS15-B73* from maize were amplified from cDNA and cloned into the sequencing vector pCR4-TOPO (Invitrogen). After complete sequencing, *ZmTPS20-Del* and *ZmTPS22-Del* were subcloned as *Bsa*I fragments into the expression vector pASK-IBA7 (IBA-GmbH, Göttingen, Germany). Before subcloning, an internal *Bsa*I restriction site in *ZmTPS22-Del* was mutated by site-directed mutagenesis as described in Köllner et al. 2006. The ORF of *ZmTPS15-B73* lacking the first 68 codons, which encode the N-terminal signal peptide, was subcloned into the expression vector pET100/D-TOPO (Invitrogen). The complete ORFs of Bradi3g14710 and Bradi3g15956 from *Brachypodium distachyon* were amplified from cDNA and cloned into the expression vector pEXP5-CT/TOPO (Invitrogen). The reconstructed ancestor sequences of clades I–III were synthesized as codon-optimized genes and subsequently cloned into the expression vector pET100/D-TOPO. The N-terminal signal peptide (33 amino acids) of the clade III ancestor was truncated before heterologous expression. All primers used for amplification, subcloning, and mutagenesis are listed in Supplemental Table S2. Sequences were deposited in GenBank (https://www.ncbi.nlm.nih.gov) with the accession numbers MT294265 (*ZmTPS20-Del*), MT294266 (*ZmTPS22-Del*), MT294267 (*ZmTPS15-B73*), MT294268 (*Bradi3g14710*), MT294269 (*Bradi3g15956*), MT294270 (clade I ancestor), MT294271 (clade II ancestor), and MT294272 (clade III ancestor). The codon-optimized sequences of the *TPS* ancestors are given in Supplemental Figure S8.

### Heterologous expression of *TPS* genes in *Escherichia coli*

For heterologous expression in *E*.* coli*, pASK-IBA7-constructs were introduced into the strain TOP10 (Invitrogen), while pET100/D-TOPO-constructs were expressed in the strain BL21 (DE3). Liquid cultures of the bacteria harboring the expression constructs were grown at 37 °C to an OD_600_ of 0.6. Expression of the recombinant proteins from pET100/D-TOPO constructs in BL21 (DE3) was induced by addition of isopropyl-b-thiogalactopyranoside to a final concentration of 1 mM, and the expression of pASK-IBA7 constructs in TOP10 cells was induced with 200 mg/l of anhydrotetracycline (IBA, Göttingen, Germany). Cultures were incubated for 20 h at 18 °C and cells were collected by centrifugation and disrupted by a 4 × 30 s treatment with a sonicator (Bandelin UW2070, Berlin, Germany) in chilled extraction buffer (50 mM Tris–HCl, pH 7.5, with 5 mM dithiothreitol and 10% (v/v) glycerol). The cell fragments were removed by centrifugation at 14,000 g and the supernatant was desalted into assay buffer (10 mM Tris–HCl, pH 7.5, 1 mM dithiothreitol, 10% (v/v) glycerol) by passage through a Econopac 10DG column (BioRad, Hercules, CA, USA).

### TPS enzyme assays

To determine the catalytic activity of the different terpene synthases and the reconstructed TPS ancestors, enzyme assays containing 40 μl of the bacterial extract and 60 µl assay buffer with 10 μM (*E*,*E*)-FPP or GPP and 10 mM MgCl_2_, in a Teflon-sealed, screw-capped 1 ml GC glass vial were performed. A SPME (solid phase microextraction) fiber consisting of 100 µm polydimethylsiloxane (Supelco, Bellefonte, PA, USA) was placed into the headspace of the vial for 0.5 h incubation at 30 °C. For analysis of the adsorbed reaction products, the SPME fiber was directly inserted into the injector of the gas chromatograph.

### Terpene synthase product analysis

Qualitative analysis of TPS enzyme products was conducted using an Agilent 6890 Series gas chromatograph coupled to an Agilent 5973 quadrupole mass selective detector (interface temp.: 270 °C; quadrupole temp.: 150 °C, source temp.: 230 °C, electron energy: 70 eV). The constituents of the product blends were separated with a DB-5MS column (Agilent, Santa Clara, CA, USA, 30 m × 0.25 mm × 0.25 µm) and helium (1 ml min^−1^) as carrier gas. The SPME fiber was injected without split at an initial oven temperature of 60 °C. The temperature was held for 2 min and then increased to 220 °C with a gradient of 7 °C min^−1^, followed by a further increase to 300 °C with 60 °C min^−1^ and a hold for 3 min. Compounds were identified by comparison of retention times and mass spectra to those of authentic standards obtained from Fluka (Seelze, Germany), Roth (Karlsruhe, Germany), Sigma (St, Louis, MO, USA), or Bedoukian (Danbury, CT, USA), or by reference spectra in the Wiley and National Institute of Standards and Technology libraries.

## Electronic supplementary material

Below is the link to the electronic supplementary material.

Supplementary file1 (PPTX 304 kb)

## Data Availability

All data generated or analyzed during this study are included in the main text or supplement of this article. Sequences of functionally characterized terpene synthases and terpene synthase ancestors are available on GenBank: MT294265-MT294272.
