# Supplementary material for: The reconstruction and biochemical characterization of ancestral genes furnish insights into the evolution of terpene synthase function in the Poaceae
Source: Plant Mol Biol. 2020 Jul 18;104(1):203–15. doi: 10.1007/s11103-020-01037-4 (PMC7417412; doi:10.1007/s11103-020-01037-4)
Supplement: Supplementary file 1 — Supplementary file1 (PPTX 304 kb) [file 11103_2020_1037_MOESM1_ESM.pptx]

## Slide 1
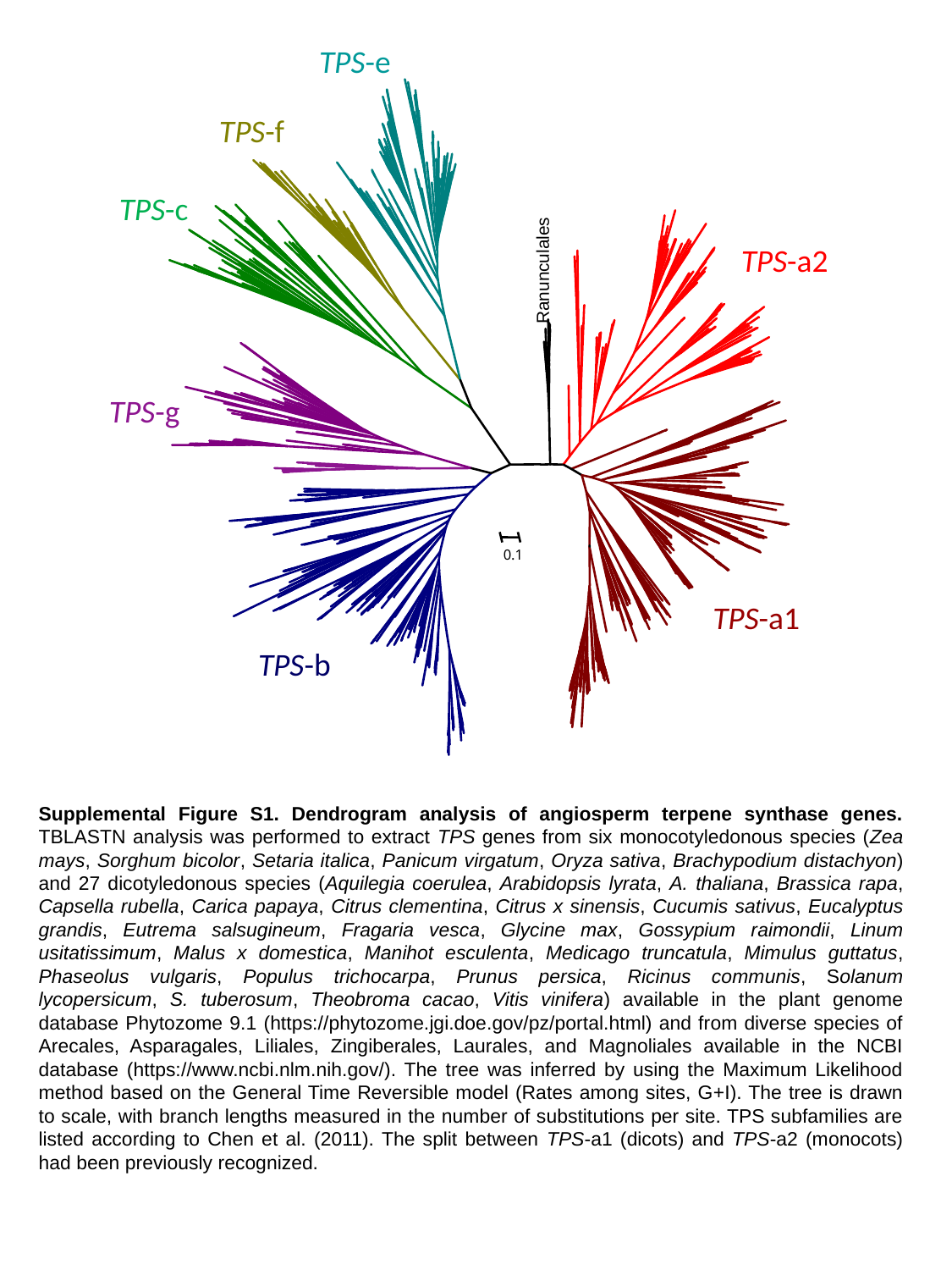

TPS-e
TPS-f
TPS-c
TPS-a2
Ranunculales
TPS-g
0.1
TPS-a1
TPS-b
Supplemental Figure S1. Dendrogram analysis of angiosperm terpene synthase genes. TBLASTN analysis was performed to extract TPS genes from six monocotyledonous species (Zea mays, Sorghum bicolor, Setaria italica, Panicum virgatum, Oryza sativa, Brachypodium distachyon) and 27 dicotyledonous species (Aquilegia coerulea, Arabidopsis lyrata, A. thaliana, Brassica rapa, Capsella rubella, Carica papaya, Citrus clementina, Citrus x sinensis, Cucumis sativus, Eucalyptus grandis, Eutrema salsugineum, Fragaria vesca, Glycine max, Gossypium raimondii, Linum usitatissimum, Malus x domestica, Manihot esculenta, Medicago truncatula, Mimulus guttatus, Phaseolus vulgaris, Populus trichocarpa, Prunus persica, Ricinus communis, Solanum lycopersicum, S. tuberosum, Theobroma cacao, Vitis vinifera) available in the plant genome database Phytozome 9.1 (https://phytozome.jgi.doe.gov/pz/portal.html) and from diverse species of Arecales, Asparagales, Liliales, Zingiberales, Laurales, and Magnoliales available in the NCBI database (https://www.ncbi.nlm.nih.gov/). The tree was inferred by using the Maximum Likelihood method based on the General Time Reversible model (Rates among sites, G+I). The tree is drawn to scale, with branch lengths measured in the number of substitutions per site. TPS subfamilies are listed according to Chen et al. (2011). The split between TPS-a1 (dicots) and TPS-a2 (monocots) had been previously recognized.

## Slide 2
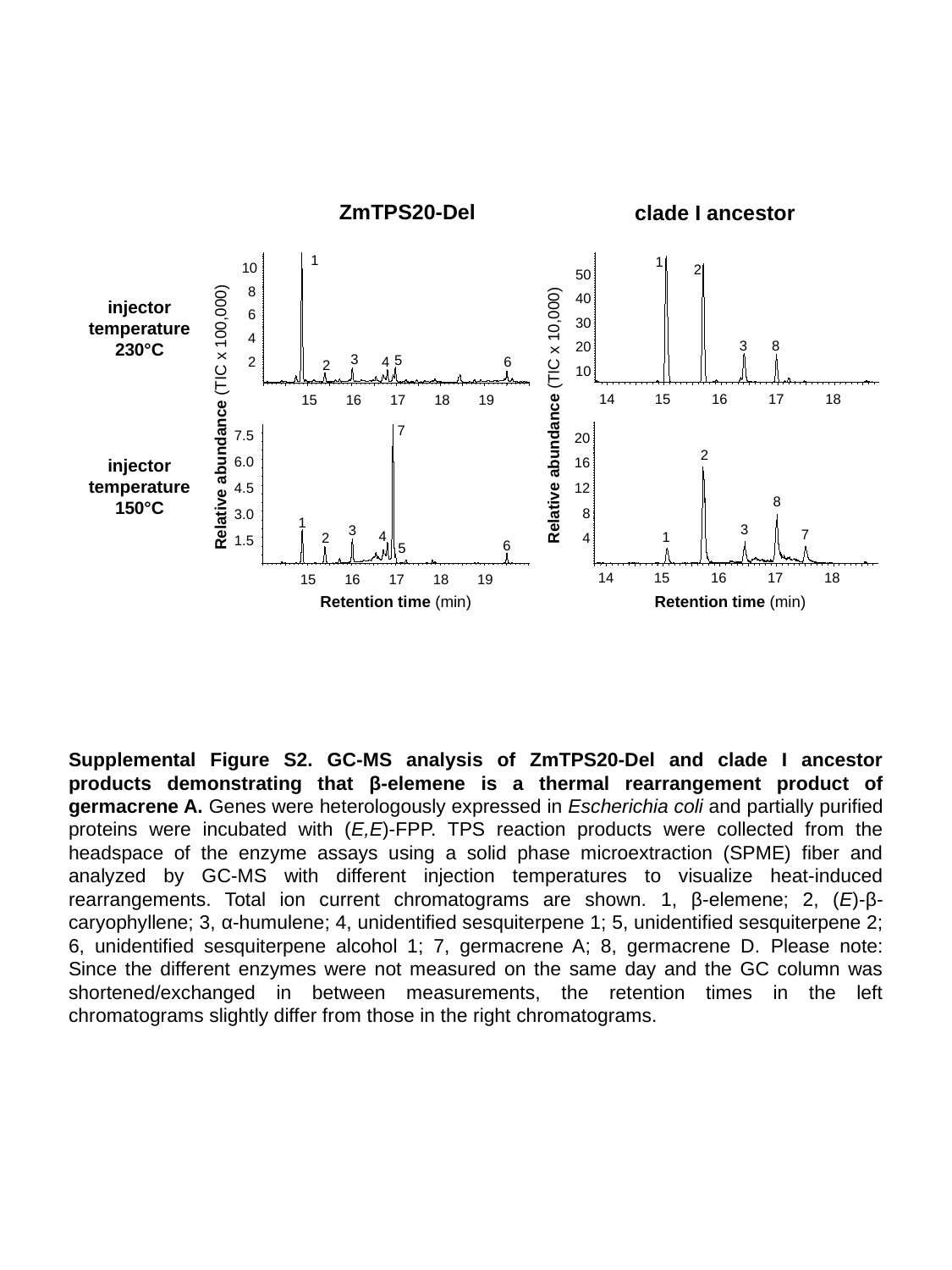

ZmTPS20-Del
clade I ancestor
1
1
2
50
40
30
3
8
20
10
15
16
17
18
14
10
8
injector
temperature
230°C
6
4
3
5
4
6
2
2
15
16
17
18
19
Relative abundance (TIC x 10,000)
Relative abundance (TIC x 100,000)
7
20
2
16
12
8
8
3
7
1
4
15
16
17
18
14
7.5
injector
temperature
150°C
6.0
4.5
3.0
1
3
4
2
6
1.5
5
15
16
17
18
19
Retention time (min)
Retention time (min)
Supplemental Figure S2. GC-MS analysis of ZmTPS20-Del and clade I ancestor products demonstrating that β-elemene is a thermal rearrangement product of germacrene A. Genes were heterologously expressed in Escherichia coli and partially purified proteins were incubated with (E,E)-FPP. TPS reaction products were collected from the headspace of the enzyme assays using a solid phase microextraction (SPME) fiber and analyzed by GC-MS with different injection temperatures to visualize heat-induced rearrangements. Total ion current chromatograms are shown. 1, β-elemene; 2, (E)-β-caryophyllene; 3, α-humulene; 4, unidentified sesquiterpene 1; 5, unidentified sesquiterpene 2; 6, unidentified sesquiterpene alcohol 1; 7, germacrene A; 8, germacrene D. Please note: Since the different enzymes were not measured on the same day and the GC column was shortened/exchanged in between measurements, the retention times in the left chromatograms slightly differ from those in the right chromatograms.

## Slide 3
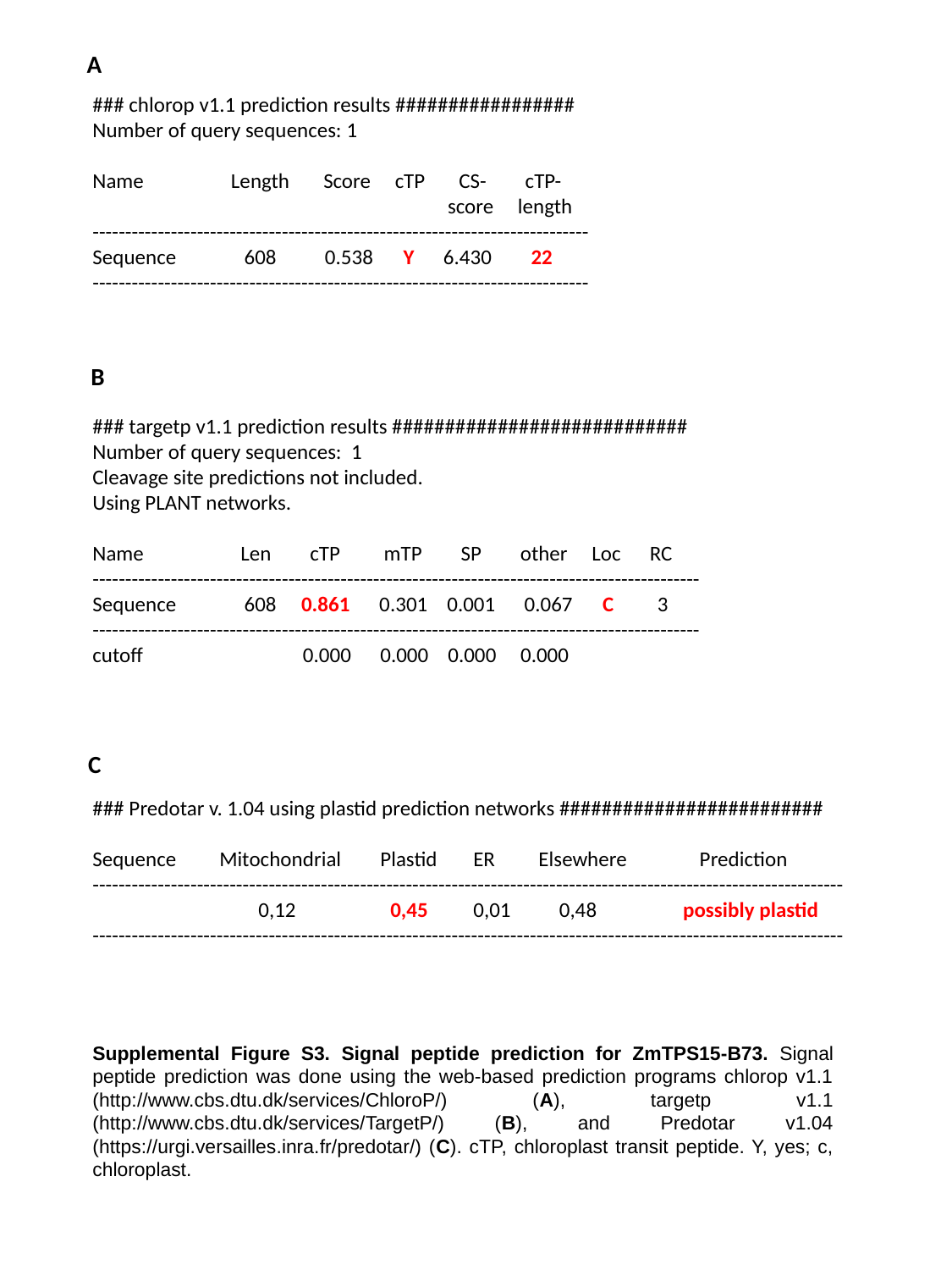

A
### chlorop v1.1 prediction results #################
Number of query sequences: 1
Name Length Score cTP CS- cTP-
 score length
----------------------------------------------------------------------------
Sequence 608 0.538 Y 6.430 22
----------------------------------------------------------------------------
B
### targetp v1.1 prediction results ############################
Number of query sequences: 1
Cleavage site predictions not included.
Using PLANT networks.
Name Len cTP mTP SP other Loc RC
---------------------------------------------------------------------------------------------
Sequence 608 0.861 0.301 0.001 0.067 C 3
---------------------------------------------------------------------------------------------
cutoff 0.000 0.000 0.000 0.000
C
### Predotar v. 1.04 using plastid prediction networks #########################
Sequence	Mitochondrial	 Plastid	ER Elsewhere Prediction
-------------------------------------------------------------------------------------------------------------------
	 0,12	 0,45	0,01 0,48	 possibly plastid
-------------------------------------------------------------------------------------------------------------------
Supplemental Figure S3. Signal peptide prediction for ZmTPS15-B73. Signal peptide prediction was done using the web-based prediction programs chlorop v1.1 (http://www.cbs.dtu.dk/services/ChloroP/) (A), targetp v1.1 (http://www.cbs.dtu.dk/services/TargetP/) (B), and Predotar v1.04 (https://urgi.versailles.inra.fr/predotar/) (C). cTP, chloroplast transit peptide. Y, yes; c, chloroplast.

## Slide 4
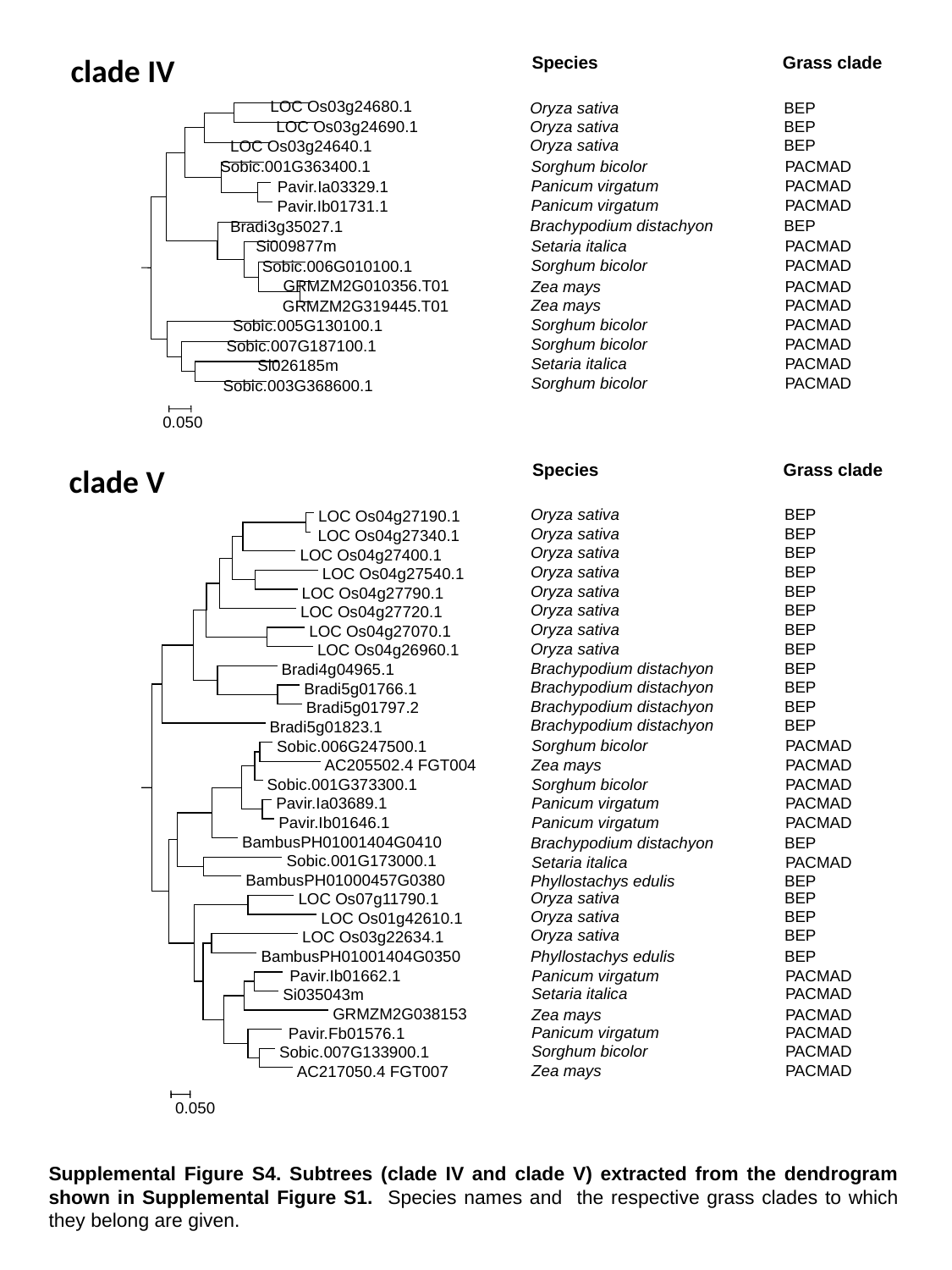

clade IV
Species Grass clade
 LOC Os03g24680.1
 LOC Os03g24690.1
 LOC Os03g24640.1
 Sobic.001G363400.1
 Pavir.Ia03329.1
 Pavir.Ib01731.1
 Bradi3g35027.1
 Si009877m
 Sobic.006G010100.1
 GRMZM2G010356.T01
 GRMZM2G319445.T01
 Sobic.005G130100.1
 Sobic.007G187100.1
 Si026185m
 Sobic.003G368600.1
0.050
Oryza sativa		BEP
Oryza sativa		BEP
Oryza sativa		BEP
Sorghum bicolor		PACMAD
Panicum virgatum	PACMAD
Panicum virgatum	PACMAD
Brachypodium distachyon	BEP
Setaria italica		PACMAD
Sorghum bicolor		PACMAD
Zea mays		PACMAD
Zea mays		PACMAD
Sorghum bicolor		PACMAD
Sorghum bicolor		PACMAD
Setaria italica		PACMAD
Sorghum bicolor		PACMAD
Species Grass clade
clade V
 LOC Os04g27190.1
 LOC Os04g27340.1
 LOC Os04g27400.1
 LOC Os04g27540.1
 LOC Os04g27790.1
 LOC Os04g27720.1
 LOC Os04g27070.1
 LOC Os04g26960.1
 Bradi4g04965.1
 Bradi5g01766.1
 Bradi5g01797.2
 Bradi5g01823.1
 Sobic.006G247500.1
 AC205502.4 FGT004
 Sobic.001G373300.1
 Pavir.Ia03689.1
 Pavir.Ib01646.1
 BambusPH01001404G0410
 Sobic.001G173000.1
 BambusPH01000457G0380
 LOC Os07g11790.1
 LOC Os01g42610.1
 LOC Os03g22634.1
 BambusPH01001404G0350
 Pavir.Ib01662.1
 Si035043m
 GRMZM2G038153
 Pavir.Fb01576.1
 Sobic.007G133900.1
 AC217050.4 FGT007
0.050
Oryza sativa		BEP
Oryza sativa		BEP
Oryza sativa		BEP
Oryza sativa		BEP
Oryza sativa		BEP
Oryza sativa		BEP
Oryza sativa		BEP
Oryza sativa		BEP
Brachypodium distachyon	BEP
Brachypodium distachyon	BEP
Brachypodium distachyon	BEP
Brachypodium distachyon	BEP
Sorghum bicolor		PACMAD
Zea mays		PACMAD
Sorghum bicolor		PACMAD
Panicum virgatum	PACMAD
Panicum virgatum	PACMAD
Brachypodium distachyon	BEP
Setaria italica		PACMAD
Phyllostachys edulis	BEP
Oryza sativa		BEP
Oryza sativa		BEP
Oryza sativa		BEP
Phyllostachys edulis	BEP
Panicum virgatum	PACMAD
Setaria italica		PACMAD
Zea mays		PACMAD
Panicum virgatum	PACMAD
Sorghum bicolor		PACMAD
Zea mays		PACMAD
Supplemental Figure S4. Subtrees (clade IV and clade V) extracted from the dendrogram shown in Supplemental Figure S1. Species names and the respective grass clades to which they belong are given.

## Slide 5
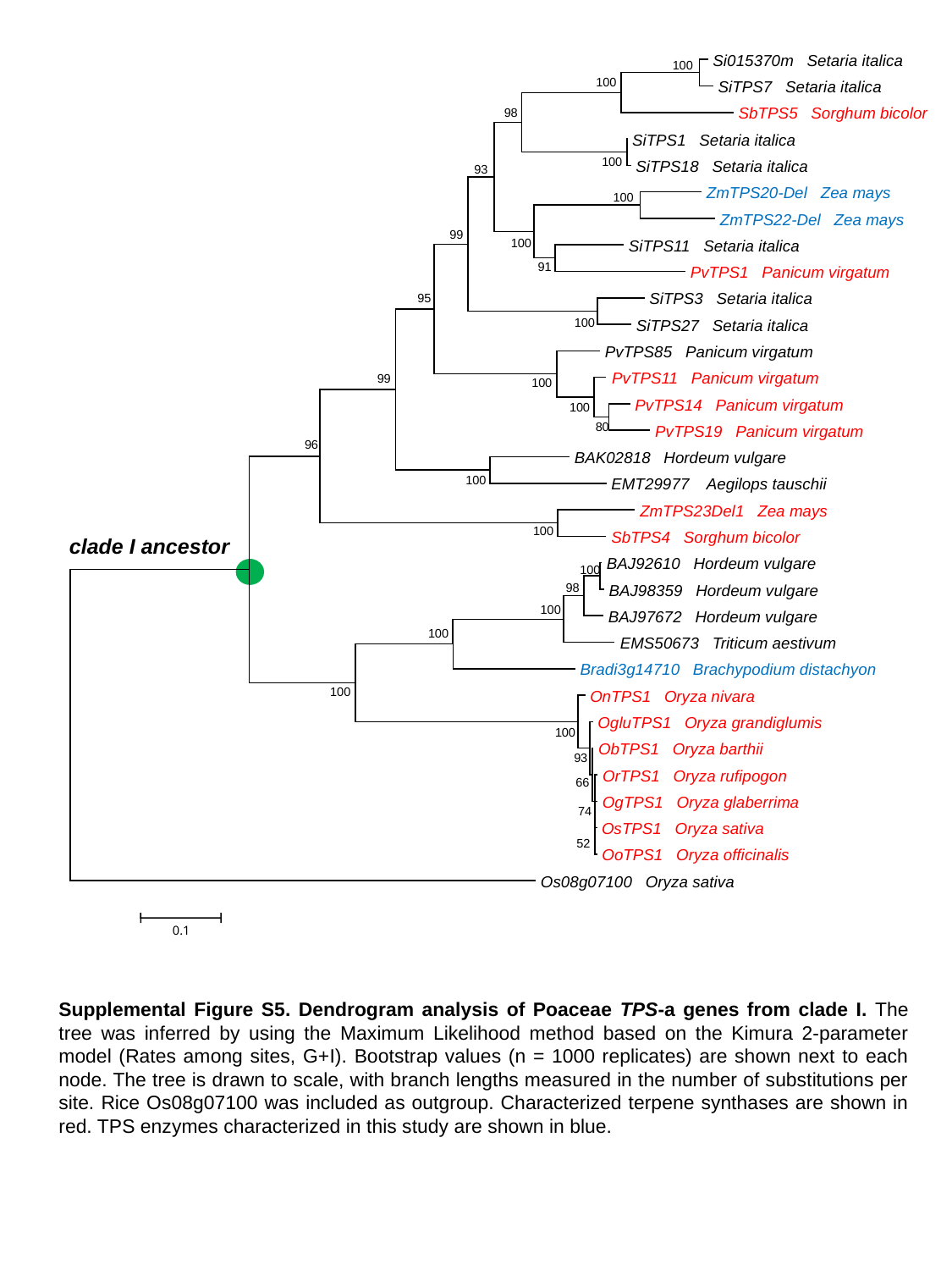

Si015370m Setaria italica
100
100
 SiTPS7 Setaria italica
 SbTPS5 Sorghum bicolor
98
 SiTPS1 Setaria italica
100
 SiTPS18 Setaria italica
93
 ZmTPS20-Del Zea mays
100
 ZmTPS22-Del Zea mays
99
100
 SiTPS11 Setaria italica
91
 PvTPS1 Panicum virgatum
 SiTPS3 Setaria italica
95
100
 SiTPS27 Setaria italica
 PvTPS85 Panicum virgatum
 PvTPS11 Panicum virgatum
99
100
 PvTPS14 Panicum virgatum
100
80
 PvTPS19 Panicum virgatum
96
 BAK02818 Hordeum vulgare
100
 EMT29977 Aegilops tauschii
 ZmTPS23Del1 Zea mays
100
clade I ancestor
 SbTPS4 Sorghum bicolor
 BAJ92610 Hordeum vulgare
100
98
 BAJ98359 Hordeum vulgare
100
 BAJ97672 Hordeum vulgare
100
 EMS50673 Triticum aestivum
 Bradi3g14710 Brachypodium distachyon
100
 OnTPS1 Oryza nivara
 OgluTPS1 Oryza grandiglumis
100
 ObTPS1 Oryza barthii
93
 OrTPS1 Oryza rufipogon
66
 OgTPS1 Oryza glaberrima
74
 OsTPS1 Oryza sativa
52
 OoTPS1 Oryza officinalis
 Os08g07100 Oryza sativa
0.1
Supplemental Figure S5. Dendrogram analysis of Poaceae TPS-a genes from clade I. The tree was inferred by using the Maximum Likelihood method based on the Kimura 2-parameter model (Rates among sites, G+I). Bootstrap values (n = 1000 replicates) are shown next to each node. The tree is drawn to scale, with branch lengths measured in the number of substitutions per site. Rice Os08g07100 was included as outgroup. Characterized terpene synthases are shown in red. TPS enzymes characterized in this study are shown in blue.

## Slide 6
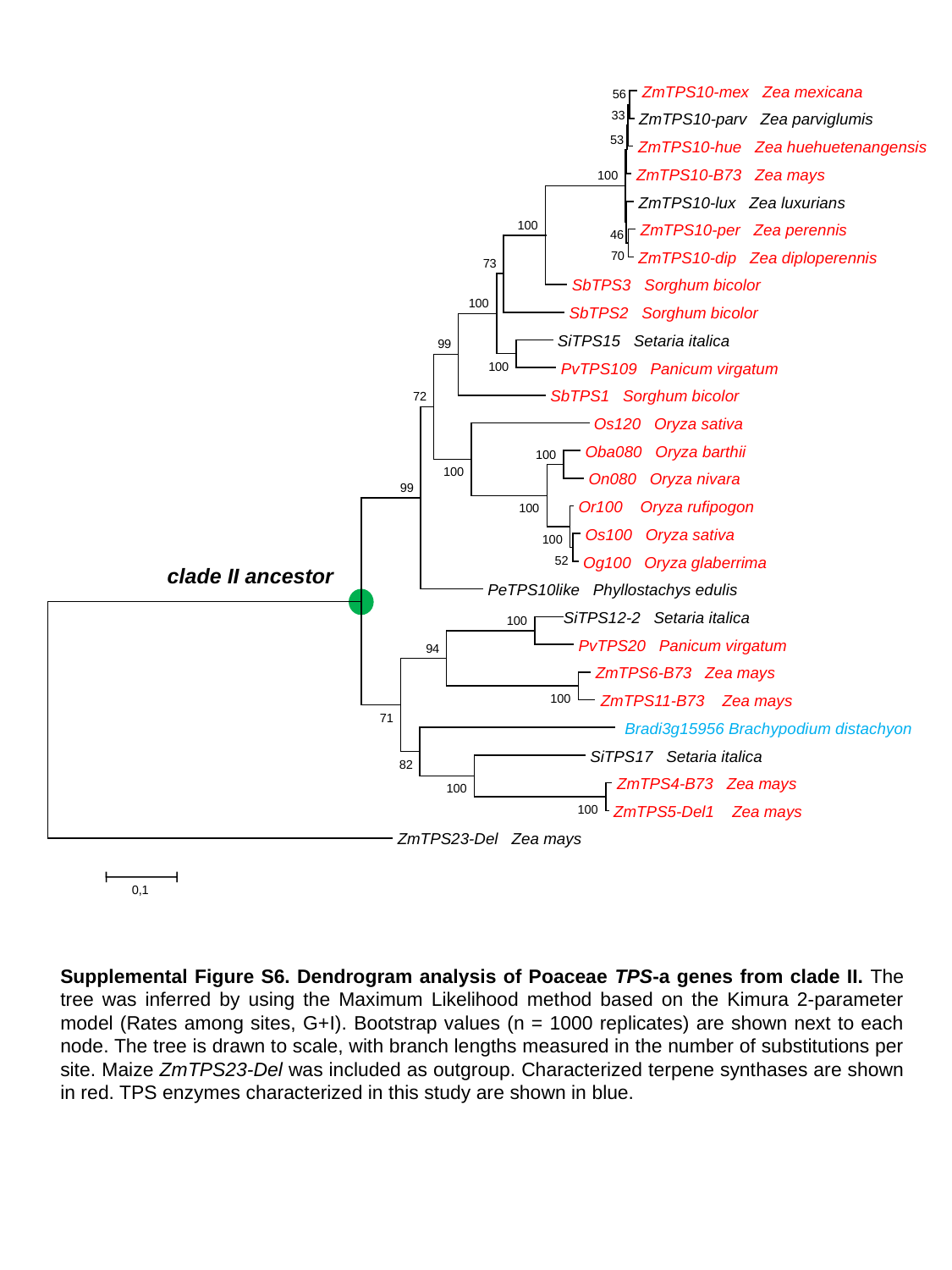

ZmTPS10-mex Zea mexicana
56
33
 ZmTPS10-parv Zea parviglumis
53
 ZmTPS10-hue Zea huehuetenangensis
 ZmTPS10-B73 Zea mays
100
 ZmTPS10-lux Zea luxurians
100
 ZmTPS10-per Zea perennis
46
 ZmTPS10-dip Zea diploperennis
70
73
 SbTPS3 Sorghum bicolor
100
 SbTPS2 Sorghum bicolor
 SiTPS15 Setaria italica
99
 PvTPS109 Panicum virgatum
100
 SbTPS1 Sorghum bicolor
72
 Os120 Oryza sativa
 Oba080 Oryza barthii
100
100
 On080 Oryza nivara
99
 Or100 Oryza rufipogon
100
 Os100 Oryza sativa
100
 Og100 Oryza glaberrima
52
clade II ancestor
 PeTPS10like Phyllostachys edulis
SiTPS12-2 Setaria italica
100
 PvTPS20 Panicum virgatum
94
 ZmTPS6-B73 Zea mays
 ZmTPS11-B73 Zea mays
100
71
 Bradi3g15956 Brachypodium distachyon
 SiTPS17 Setaria italica
82
 ZmTPS4-B73 Zea mays
100
 ZmTPS5-Del1 Zea mays
100
 ZmTPS23-Del Zea mays
0,1
Supplemental Figure S6. Dendrogram analysis of Poaceae TPS-a genes from clade II. The tree was inferred by using the Maximum Likelihood method based on the Kimura 2-parameter model (Rates among sites, G+I). Bootstrap values (n = 1000 replicates) are shown next to each node. The tree is drawn to scale, with branch lengths measured in the number of substitutions per site. Maize ZmTPS23-Del was included as outgroup. Characterized terpene synthases are shown in red. TPS enzymes characterized in this study are shown in blue.

## Slide 7
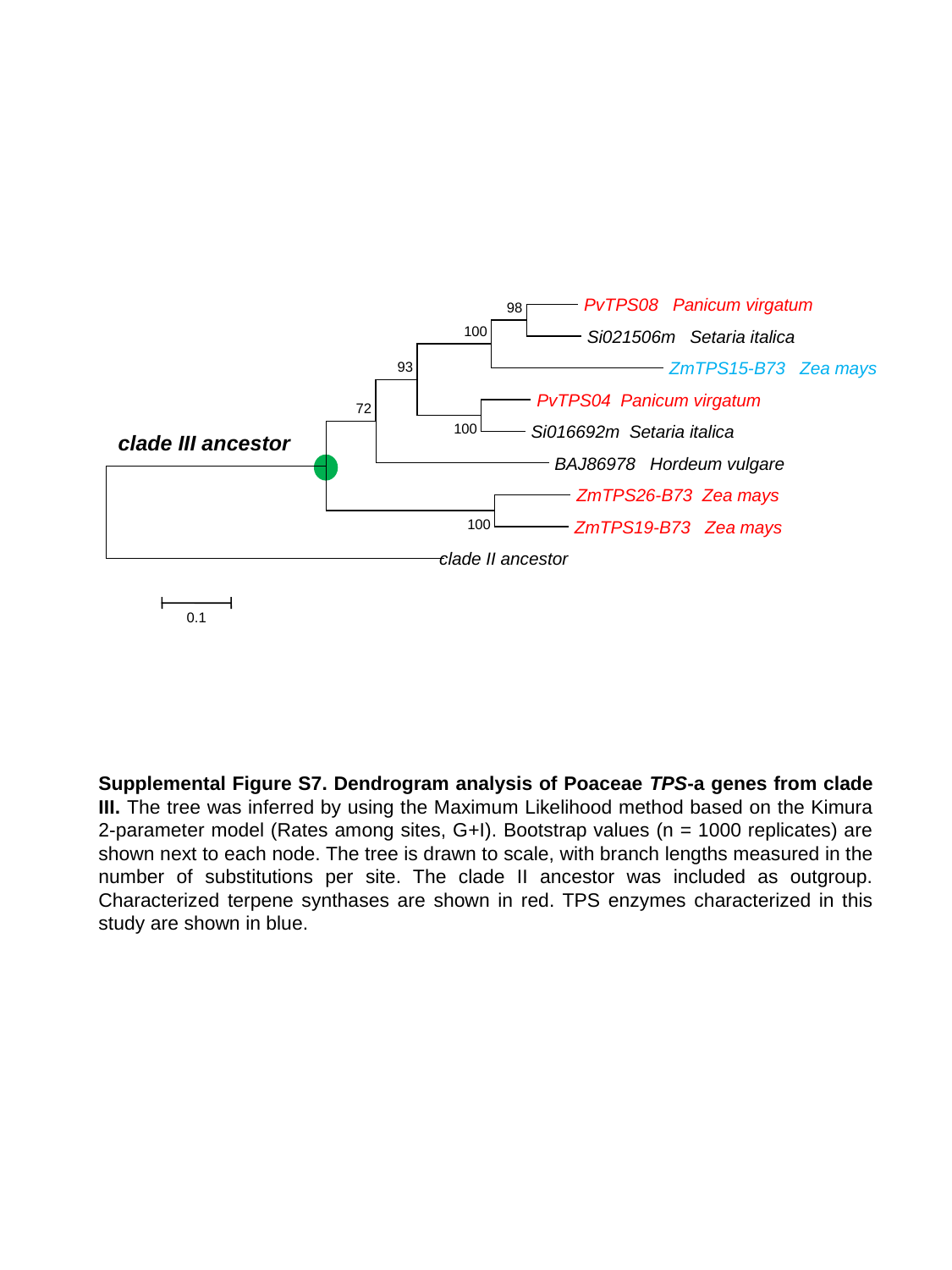

PvTPS08 Panicum virgatum
98
100
 Si021506m Setaria italica
 ZmTPS15-B73 Zea mays
93
 PvTPS04 Panicum virgatum
72
100
 Si016692m Setaria italica
clade III ancestor
 BAJ86978 Hordeum vulgare
 ZmTPS26-B73 Zea mays
100
 ZmTPS19-B73 Zea mays
 clade II ancestor
0.1
Supplemental Figure S7. Dendrogram analysis of Poaceae TPS-a genes from clade III. The tree was inferred by using the Maximum Likelihood method based on the Kimura 2-parameter model (Rates among sites, G+I). Bootstrap values (n = 1000 replicates) are shown next to each node. The tree is drawn to scale, with branch lengths measured in the number of substitutions per site. The clade II ancestor was included as outgroup. Characterized terpene synthases are shown in red. TPS enzymes characterized in this study are shown in blue.

## Slide 8
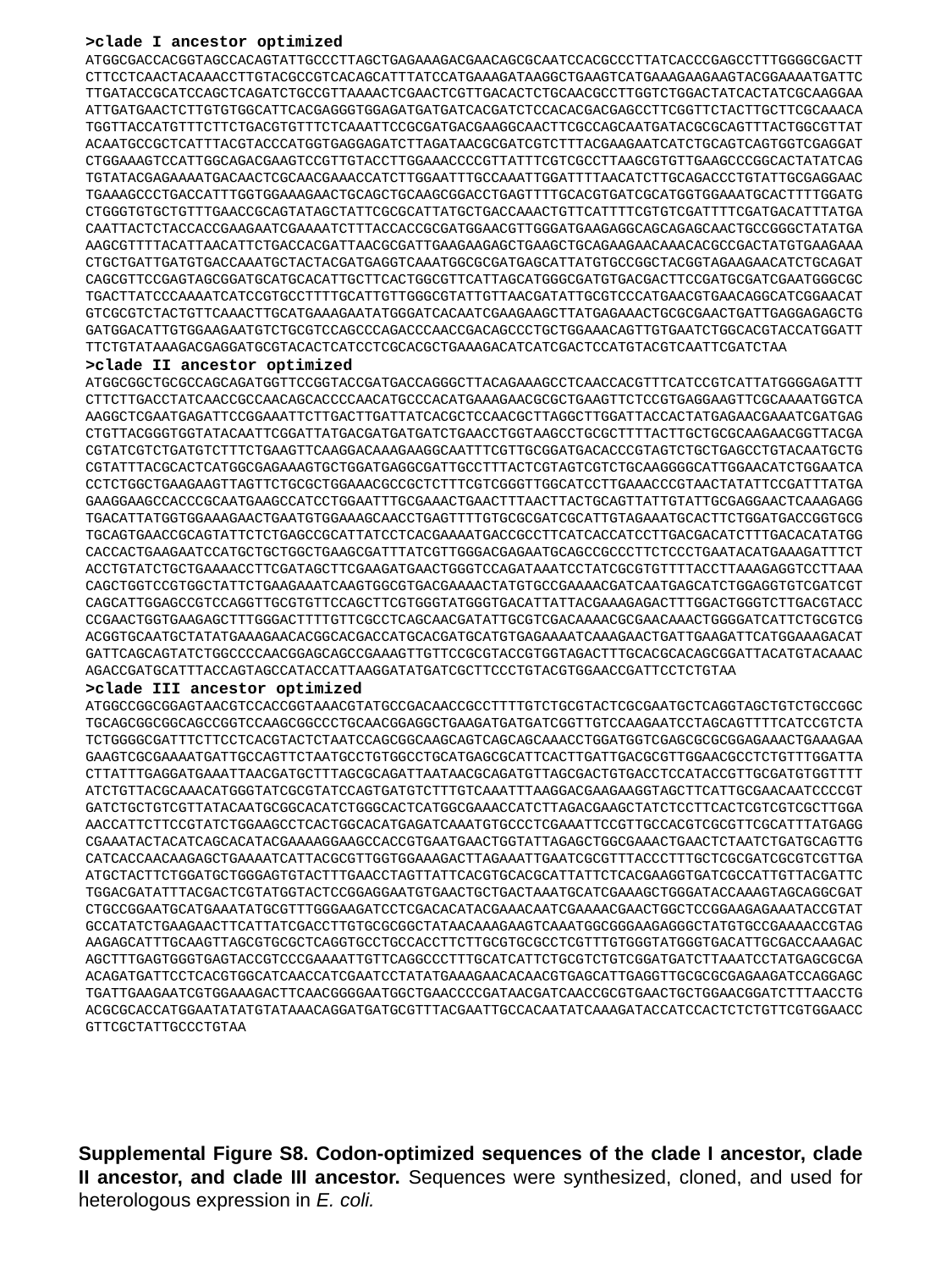

>clade I ancestor optimized
ATGGCGACCACGGTAGCCACAGTATTGCCCTTAGCTGAGAAAGACGAACAGCGCAATCCACGCCCTTATCACCCGAGCCTTTGGGGCGACTTCTTCCTCAACTACAAACCTTGTACGCCGTCACAGCATTTATCCATGAAAGATAAGGCTGAAGTCATGAAAGAAGAAGTACGGAAAATGATTCTTGATACCGCATCCAGCTCAGATCTGCCGTTAAAACTCGAACTCGTTGACACTCTGCAACGCCTTGGTCTGGACTATCACTATCGCAAGGAAATTGATGAACTCTTGTGTGGCATTCACGAGGGTGGAGATGATGATCACGATCTCCACACGACGAGCCTTCGGTTCTACTTGCTTCGCAAACATGGTTACCATGTTTCTTCTGACGTGTTTCTCAAATTCCGCGATGACGAAGGCAACTTCGCCAGCAATGATACGCGCAGTTTACTGGCGTTATACAATGCCGCTCATTTACGTACCCATGGTGAGGAGATCTTAGATAACGCGATCGTCTTTACGAAGAATCATCTGCAGTCAGTGGTCGAGGATCTGGAAAGTCCATTGGCAGACGAAGTCCGTTGTACCTTGGAAACCCCGTTATTTCGTCGCCTTAAGCGTGTTGAAGCCCGGCACTATATCAGTGTATACGAGAAAATGACAACTCGCAACGAAACCATCTTGGAATTTGCCAAATTGGATTTTAACATCTTGCAGACCCTGTATTGCGAGGAACTGAAAGCCCTGACCATTTGGTGGAAAGAACTGCAGCTGCAAGCGGACCTGAGTTTTGCACGTGATCGCATGGTGGAAATGCACTTTTGGATGCTGGGTGTGCTGTTTGAACCGCAGTATAGCTATTCGCGCATTATGCTGACCAAACTGTTCATTTTCGTGTCGATTTTCGATGACATTTATGACAATTACTCTACCACCGAAGAATCGAAAATCTTTACCACCGCGATGGAACGTTGGGATGAAGAGGCAGCAGAGCAACTGCCGGGCTATATGAAAGCGTTTTACATTAACATTCTGACCACGATTAACGCGATTGAAGAAGAGCTGAAGCTGCAGAAGAACAAACACGCCGACTATGTGAAGAAACTGCTGATTGATGTGACCAAATGCTACTACGATGAGGTCAAATGGCGCGATGAGCATTATGTGCCGGCTACGGTAGAAGAACATCTGCAGATCAGCGTTCCGAGTAGCGGATGCATGCACATTGCTTCACTGGCGTTCATTAGCATGGGCGATGTGACGACTTCCGATGCGATCGAATGGGCGCTGACTTATCCCAAAATCATCCGTGCCTTTTGCATTGTTGGGCGTATTGTTAACGATATTGCGTCCCATGAACGTGAACAGGCATCGGAACATGTCGCGTCTACTGTTCAAACTTGCATGAAAGAATATGGGATCACAATCGAAGAAGCTTATGAGAAACTGCGCGAACTGATTGAGGAGAGCTGGATGGACATTGTGGAAGAATGTCTGCGTCCAGCCCAGACCCAACCGACAGCCCTGCTGGAAACAGTTGTGAATCTGGCACGTACCATGGATTTTCTGTATAAAGACGAGGATGCGTACACTCATCCTCGCACGCTGAAAGACATCATCGACTCCATGTACGTCAATTCGATCTAA
>clade II ancestor optimized
ATGGCGGCTGCGCCAGCAGATGGTTCCGGTACCGATGACCAGGGCTTACAGAAAGCCTCAACCACGTTTCATCCGTCATTATGGGGAGATTTCTTCTTGACCTATCAACCGCCAACAGCACCCCAACATGCCCACATGAAAGAACGCGCTGAAGTTCTCCGTGAGGAAGTTCGCAAAATGGTCAAAGGCTCGAATGAGATTCCGGAAATTCTTGACTTGATTATCACGCTCCAACGCTTAGGCTTGGATTACCACTATGAGAACGAAATCGATGAGCTGTTACGGGTGGTATACAATTCGGATTATGACGATGATGATCTGAACCTGGTAAGCCTGCGCTTTTACTTGCTGCGCAAGAACGGTTACGACGTATCGTCTGATGTCTTTCTGAAGTTCAAGGACAAAGAAGGCAATTTCGTTGCGGATGACACCCGTAGTCTGCTGAGCCTGTACAATGCTGCGTATTTACGCACTCATGGCGAGAAAGTGCTGGATGAGGCGATTGCCTTTACTCGTAGTCGTCTGCAAGGGGCATTGGAACATCTGGAATCACCTCTGGCTGAAGAAGTTAGTTCTGCGCTGGAAACGCCGCTCTTTCGTCGGGTTGGCATCCTTGAAACCCGTAACTATATTCCGATTTATGAGAAGGAAGCCACCCGCAATGAAGCCATCCTGGAATTTGCGAAACTGAACTTTAACTTACTGCAGTTATTGTATTGCGAGGAACTCAAAGAGGTGACATTATGGTGGAAAGAACTGAATGTGGAAAGCAACCTGAGTTTTGTGCGCGATCGCATTGTAGAAATGCACTTCTGGATGACCGGTGCGTGCAGTGAACCGCAGTATTCTCTGAGCCGCATTATCCTCACGAAAATGACCGCCTTCATCACCATCCTTGACGACATCTTTGACACATATGGCACCACTGAAGAATCCATGCTGCTGGCTGAAGCGATTTATCGTTGGGACGAGAATGCAGCCGCCCTTCTCCCTGAATACATGAAAGATTTCTACCTGTATCTGCTGAAAACCTTCGATAGCTTCGAAGATGAACTGGGTCCAGATAAATCCTATCGCGTGTTTTACCTTAAAGAGGTCCTTAAACAGCTGGTCCGTGGCTATTCTGAAGAAATCAAGTGGCGTGACGAAAACTATGTGCCGAAAACGATCAATGAGCATCTGGAGGTGTCGATCGTCAGCATTGGAGCCGTCCAGGTTGCGTGTTCCAGCTTCGTGGGTATGGGTGACATTATTACGAAAGAGACTTTGGACTGGGTCTTGACGTACCCCGAACTGGTGAAGAGCTTTGGGACTTTTGTTCGCCTCAGCAACGATATTGCGTCGACAAAACGCGAACAAACTGGGGATCATTCTGCGTCGACGGTGCAATGCTATATGAAAGAACACGGCACGACCATGCACGATGCATGTGAGAAAATCAAAGAACTGATTGAAGATTCATGGAAAGACATGATTCAGCAGTATCTGGCCCCAACGGAGCAGCCGAAAGTTGTTCCGCGTACCGTGGTAGACTTTGCACGCACAGCGGATTACATGTACAAACAGACCGATGCATTTACCAGTAGCCATACCATTAAGGATATGATCGCTTCCCTGTACGTGGAACCGATTCCTCTGTAA
>clade III ancestor optimized
ATGGCCGGCGGAGTAACGTCCACCGGTAAACGTATGCCGACAACCGCCTTTTGTCTGCGTACTCGCGAATGCTCAGGTAGCTGTCTGCCGGCTGCAGCGGCGGCAGCCGGTCCAAGCGGCCCTGCAACGGAGGCTGAAGATGATGATCGGTTGTCCAAGAATCCTAGCAGTTTTCATCCGTCTATCTGGGGCGATTTCTTCCTCACGTACTCTAATCCAGCGGCAAGCAGTCAGCAGCAAACCTGGATGGTCGAGCGCGCGGAGAAACTGAAAGAAGAAGTCGCGAAAATGATTGCCAGTTCTAATGCCTGTGGCCTGCATGAGCGCATTCACTTGATTGACGCGTTGGAACGCCTCTGTTTGGATTACTTATTTGAGGATGAAATTAACGATGCTTTAGCGCAGATTAATAACGCAGATGTTAGCGACTGTGACCTCCATACCGTTGCGATGTGGTTTTATCTGTTACGCAAACATGGGTATCGCGTATCCAGTGATGTCTTTGTCAAATTTAAGGACGAAGAAGGTAGCTTCATTGCGAACAATCCCCGTGATCTGCTGTCGTTATACAATGCGGCACATCTGGGCACTCATGGCGAAACCATCTTAGACGAAGCTATCTCCTTCACTCGTCGTCGCTTGGAAACCATTCTTCCGTATCTGGAAGCCTCACTGGCACATGAGATCAAATGTGCCCTCGAAATTCCGTTGCCACGTCGCGTTCGCATTTATGAGGCGAAATACTACATCAGCACATACGAAAAGGAAGCCACCGTGAATGAACTGGTATTAGAGCTGGCGAAACTGAACTCTAATCTGATGCAGTTGCATCACCAACAAGAGCTGAAAATCATTACGCGTTGGTGGAAAGACTTAGAAATTGAATCGCGTTTACCCTTTGCTCGCGATCGCGTCGTTGAATGCTACTTCTGGATGCTGGGAGTGTACTTTGAACCTAGTTATTCACGTGCACGCATTATTCTCACGAAGGTGATCGCCATTGTTACGATTCTGGACGATATTTACGACTCGTATGGTACTCCGGAGGAATGTGAACTGCTGACTAAATGCATCGAAAGCTGGGATACCAAAGTAGCAGGCGATCTGCCGGAATGCATGAAATATGCGTTTGGGAAGATCCTCGACACATACGAAACAATCGAAAACGAACTGGCTCCGGAAGAGAAATACCGTATGCCATATCTGAAGAACTTCATTATCGACCTTGTGCGCGGCTATAACAAAGAAGTCAAATGGCGGGAAGAGGGCTATGTGCCGAAAACCGTAGAAGAGCATTTGCAAGTTAGCGTGCGCTCAGGTGCCTGCCACCTTCTTGCGTGCGCCTCGTTTGTGGGTATGGGTGACATTGCGACCAAAGACAGCTTTGAGTGGGTGAGTACCGTCCCGAAAATTGTTCAGGCCCTTTGCATCATTCTGCGTCTGTCGGATGATCTTAAATCCTATGAGCGCGAACAGATGATTCCTCACGTGGCATCAACCATCGAATCCTATATGAAAGAACACAACGTGAGCATTGAGGTTGCGCGCGAGAAGATCCAGGAGCTGATTGAAGAATCGTGGAAAGACTTCAACGGGGAATGGCTGAACCCCGATAACGATCAACCGCGTGAACTGCTGGAACGGATCTTTAACCTGACGCGCACCATGGAATATATGTATAAACAGGATGATGCGTTTACGAATTGCCACAATATCAAAGATACCATCCACTCTCTGTTCGTGGAACCGTTCGCTATTGCCCTGTAA
Supplemental Figure S8. Codon-optimized sequences of the clade I ancestor, clade II ancestor, and clade III ancestor. Sequences were synthesized, cloned, and used for heterologous expression in E. coli.
